# Domestication of *Oryza* species eco-evolutionarily shapes bacterial and fungal communities in rice seed

**DOI:** 10.1186/s40168-020-00805-0

**Published:** 2020-02-14

**Authors:** Hyun Kim, Kiseok Keith Lee, Jongbum Jeon, William Anthony Harris, Yong-Hwan Lee

**Affiliations:** 1 Department of Agricultural Biotechnology, Seoul National University, Seoul, 08826 Korea; 2 Interdisciplinary Program in Agricultural Genomics, Seoul National University, Seoul, 08826 Korea; 3 Center for Fungal Genetic Resources, Seoul National University, Seoul, 08826 Korea; 4 Plant Immunity Research Center, Seoul National University, Seoul, 08826 Korea; 5 Research Institute of Agriculture and Life Sciences, Seoul National University, Seoul, 08826 Korea

## Abstract

**Background:**

Plant-associated microbiomes, which are shaped by host and environmental factors, support their hosts by providing nutrients and attenuating abiotic and biotic stresses. Although host genetic factors involved in plant growth and immunity are known to shape compositions of microbial communities, the effects of host evolution on microbial communities are not well understood.

**Results:**

We show evidence that both host speciation and domestication shape seed bacterial and fungal community structures. Genome types of rice contributed to compositional variations of both communities, showing a significant phylosymbiosis with microbial composition. Following the domestication, abundance inequality of bacterial and fungal communities also commonly increased. However, composition of bacterial community was relatively conserved, whereas fungal membership was dramatically changed. These domestication effects were further corroborated when analyzed by a random forest model. With these changes, hub taxa of inter-kingdom networks were also shifted from fungi to bacteria by domestication. Furthermore, maternal inheritance of microbiota was revealed as a major path of microbial transmission across generations.

**Conclusions:**

Our findings show that evolutionary processes stochastically affect overall composition of microbial communities, whereas dramatic changes in environments during domestication contribute to assembly of microbiotas in deterministic ways in rice seed. This study further provides new insights on host evolution and microbiome, the starting point of the holobiome of plants, microbial communities, and surrounding environments.

**Video Abtract.**

## Background

**Fig. 1:**
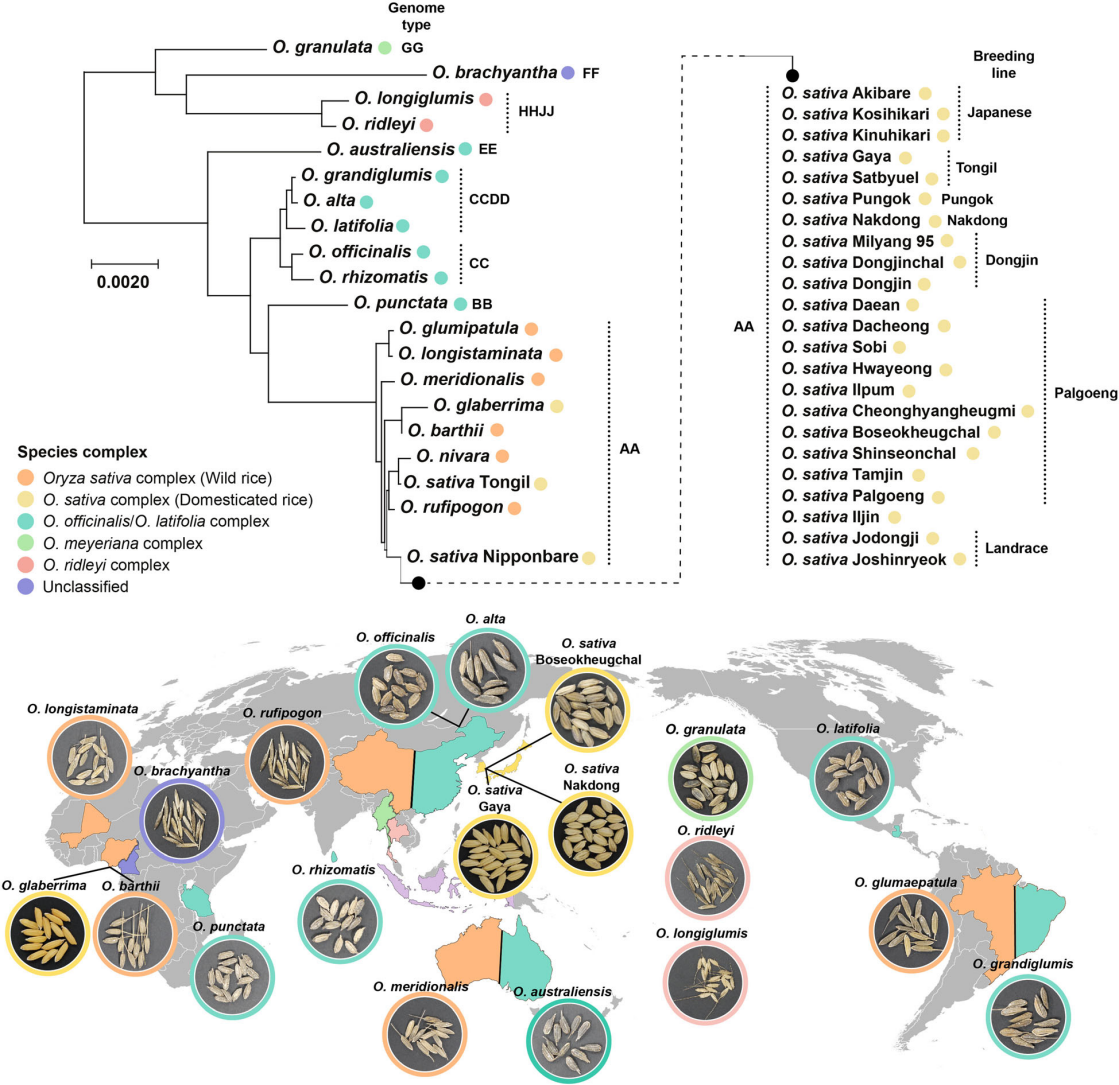
Phylogenetic tree of samples and geographic distribution. The phylogenetic tree of *Oryza* genotypes was constructed based on the chloroplast full sequence acquired from NCBI. RAxML program was used to draw a Maximum Likelihood (ML) tree with 1000 bootstraps. *O*. *sativa* cultivars were added separately to the tree to fully show the seed samples regardless of phylogenetic distance. The map indicates the diverse worldwide distribution of wild and domesticated *Oryza* spp.

The evolution of life on Earth is driven by natural selection, biased mutation, genetic drift, genetic hitchhiking, and gene flow. Regardless of plants, animals, or microorganisms, it has been ongoing for millions of years. Unlike the majority of organisms, crop plants have undergone a distinct evolutionary process called domestication. Plant domestication began ~ 12,000 years ago and 353 food crop plants including rice, wheat, barley, potato, and tomato have undergone domestication [1]. Most crop plants have been selected and been bred for better yield and quality by anthropogenic intervention. In rice, the evolution spans about 15 million years [2]. In the genus *Oryza*, there are 22 wild relatives which are distributed in Asia, Africa, Australia, and America (Fig. 1). Polyploidization and other evolutionary events contribute to speciation of *Oryza* species [3]. With the speciation, 8000–9000 years ago, *O*. *sativa* subsp. *japonica*, *O*. *sativa* subsp. *indica*, and *O*. *glaberrima* were domesticated from the wild relatives, *O*. *rufipogon*, *O*. *nivara*, and *O*. *barthii*, respectively [2]. These domesticated rice species have been further diversified by breeding to acquire desirable agronomic traits.

The phenotypes of the humans, animals, and plants are determined not only by their own genetic makeups but by their associated microbial communities. Host-associated microbial communities show significant impacts on host physiology, developments, and even fitness. Developmental stages and physiological status of host plants also can shape associated microbiotas. Previous reports on plant microbiotas suggested that bacterial communities associated with rhizosphere, root endosphere, and leaves are assembled by both host- and environment-driven factors [4, 5]. Among these factors, effects of host evolution and domestication are shaping factors on bacterial communities in the root system [4, 6–8]. However, these studies did not cover fungal communities and were conducted using only limited numbers of wild and domesticated plants. Above all, considering that root microbiotas are dynamically changed over time and by environmental conditions, the impact of evolutionary factors can be determined only at the specific time points. This limited time-scale is the obstacle for understanding evolutionary relationships of host plants and their microbiomes.

Plant seeds hold the key to solving these limitations and to answering the questions about the evolution of the microbial communities. Once the seed microbial community is encapsulated in the seed coat, it is less susceptible to exterior changes compared to other plant compartments, such as the rhizosphere. This stability enables the seed microbial community to be inherited, known as vertical transmission [9, 10], making it a prime model for investigating changes on an evolutionary timeline. The stability of plant seeds provides an invaluable opportunity to examine the effect of evolution on associated microbiota by natural and artificial selections.

Here, we report the effects of the speciation and domestication of rice on the composition of bacterial and fungal communities using 43 rice accessions (17 wild and 26 domesticated rice). Our findings suggest that seed microbiotas are more affected by stochastic factors during host speciation but domestication contributed to community assembly in a more deterministic way by dramatic changes in host and environmental factors.

## Materials and methods

### Rice seeds

Sixteen accessions of wild rice used in this study were obtained from International Rice Research Institute (IRRI), Philippines (Additional file 2). Grains of 27 rice accessions (one wild and 26 domesticated rice) were obtained from the National Agrodiversity Center in National Institute of Agricultural Sciences, Korea (Additional file 2). All rice accessions were stored at 4 °C until DNA extraction.

### Construction of phylogenetic tree of rice

A phylogenetic tree was constructed based on the chloroplast genome sequences from NCBI. RAxML program was used to construct maximum likelihood (ML) tree with 1000 bootstraps. The best tree was fitted with GTRCAT model, and rooted to *Oryza brachyantha* and *Oryza granulata* clades. Then, the phylogenetic tree was merged with other domesticated cultivars in *O*. *sativa* not by phylogenetic order but grouped by breeding history.

### DNA extraction from seeds

Before extracting the DNAs, surface sterilization was conducted by sequential treatments of 70% ethanol and 2% sodium hypochlorite (NaOCl) [11]. Each replicate consisted of three grains. The grains were ground using a homogenizer (SKMILL-200, Genomic Base, Korea). To prevent denaturation of DNAs, all tubes were stored in liquid nitrogen. The ground seeds were transferred to Lysing Matrix E tubes provided in the FastDNA SPIN Kit for Soil (MP Biomedicals, USA). The DNAs were extracted following the instructions of the manufacturer. All DNA samples were quality checked and the concentration was quantified by NanoDrop™ spectrophotometers (Thermo Scientific™, USA). The extracted DNAs were stored at − 20 °C until amplicons were generated.

### PCR amplification and sequencing

16S rRNA and internal transcribed spacer (ITS) amplicons were generated in a two-step PCR amplification protocol. V4 regions of bacterial 16S ribosomal RNA (16S rRNA) genes were amplified with universal 515F and 806R PCR primers [12] (Additional file 1: Table S2). To reduce plant mitochondrial and plastid DNA contamination, peptide nucleic acid PCR blockers (PNA clamps) were added during the first PCR [13] (Additional file 1: Table S3). The fungal ITS2 regions of the 18S ribosomal RNA genes were amplified by ITS3 and ITS4 PCR primers [14]. Each sample was amplified in triplicate in a 25 μl reaction tube containing 12.5 μl of 2× PCR i-StarTaq™ Master mix solution (Intron Biotechnology, Korea), 0.4 μM for each forward and reverse primers, 0.8 μM of diluted DNA template and peptide nucleic acid (PNA) clamps for chloroplast (pPNA), and mitochondria (mPNA) at 0.75 μM each. For the ITS libraries, the conditions were the same except the PNA clamps were not included. PCR was performed using the following program, initial denaturing at 98 °C for 3 min, followed by 25 cycles of denaturing at 98 °C for 10 s, PNA annealing at 78 °C for 10 s, primmer annealing at 55 °C for 30 s, and extension at 72 °C for 60 s. For ITS PCR amplification, the program was the same but without the PNA annealing step. Each library was accompanied by negative PCR controls to ensure that the reagents were free of contaminant DNA. Amplicon replicates were pooled, then purified using the MEGAquick-spin™ Plus DNA Purification Kit (Intron Biotechnology, Korea) with an additional ethanol clean-up step to remove unused PCR reagents and resulting primer dimers. Secondly, the PCR was done with the Nextera XT Index Kit (Illumina, USA). DNA templates were diluted to equal concentrations after being measured by the Infinite 200 pro (TECAN, Switzerland). The libraries were then pooled into equal concentrations into a single library and concentrated using AMPure beads (Beckman Coulter, USA). The pooled library then went through a final gel purification stage to remove any remaining unwanted PCR products. Pooled libraries were sequenced using the Illumina MiSeq platform with 2 × 300 base pair read length. The sequencing was done in the National Instrumentation Center for Environmental Management (NICEM) at Seoul National University, Korea.

### Processing and filtering of sequences

After demultiplexing, the resulting sequences were merged with PEAR [15] and then quality filtered with the DADA2 plugin in the QIIME2 (version 2018.6) pipeline [16]. The high-quality sequences were clustered into operational taxonomic units (OTUs) using the open reference vsearch algorithm (vsearch cluster-features-open-reference) [17] against the Silva 99% OTU representative sequence database (v132, April 2018) [18] and then assembled into an OTU table. Bacterial OTUs were chimera filtered using the vsearch uchime-denovo algorithm [19]. Fungal OTUs were checked for chimeric sequences using Uchime-ref algorithm against the dedicated chimera detection ITS2 database (June 2017 version) [20]. The taxonomy of the non-chimeric OTUs was assigned using Naïve Bayes algorithm implemented in the q2-feature-classifier prefitted to the Silva database for V4 region of 16S rRNA regions [21]. For the ITS2 region, taxonomy assignment was done with q2-feature-classifier prefitted to UNITE database (UNITE_ver7_dynamic of Jan 2017) [22]. Bacterial sequences ranging from 200 to 300 bp long and fungal sequences ranging from 100 to 490 bp long were used for further analyses. The OTU table was imported to R by the phyloseq package [23] for further analysis. Sequences from host DNA and OTUs unassigned at the kingdom-level were removed (bacterial OTU: orders “*Chloroplast*” and “*Rickettsiales*”; fungal OTU: kingdoms “*Unassigned*”, “*Chromista*”, and “*Plantae*”). OTUs detected from negative samples (false-positive OTUs) were removed from the OTU profiles of the seed samples (Additional file 3). OTUs annotated as “kingdom *Fungi*” but unidentified at the phylum level were subjected to a BLASTN search and those whose top 10 blast hits were annotated as rice ITS sequences were removed (Additional file 4). Low-abundance OTUs were eliminated if they had less than five read counts across all the samples. This process reduced the total OTU count from 390 to 364 taxa in bacteria and from 493 to 356 taxa in fungi. The resulting 364 bacterial taxa and 356 fungal taxa were used for further analysis.

### Statistical analyses and visualization

Unless otherwise stated, all statistical analyses were performed using R version 3.4.4 [24] and statistical significance was determined at ɑ = 0.05, where appropriate, the statistical significance was corrected for multiple hypothesis testing using the false discovery rate (FDR) method. The OTU table was normalized by cumulative-sum scaling (CSS) and log-transformation by cumNorm() from the R package metagenomeSeq (v. 3.8) [25] (Additional file 1: Figure S1). Since rarefying to insufficient sequence depths could lose information in high depth samples, a Hellinger transformation was done when calculating alpha diversity and plotting the Lorenz curve [26]. Shannon and Simpson indices were calculated using the diversity() in the R package Vegan (v2.5-3) [27]. Wilcoxon rank-sum test, one-way ANOVA, and Tukey HSD were all performed in R. The Lorenz curve, which is the cumulative share of total abundance plotted against the cumulative percentage of OTUs from the lowest to highest abundance, was visualized using R package ineq (v0.2-13) [28]. The index of inequality (Gini coefficient) was measured by calculating the area between the Lorenz curve and the diagonal line divided by the area of the lower left triangle as the denominator (the larger the Gini coefficient, the larger the inequality). The Bray–Curtis dissimilarity matrix was calculated to build both unconstrained and constrained principal coordinate analyses. The constrained analysis of principal coordinates (CAP) was constrained by rice genome type, domestication status, and breeding line, respectively, using the function capscale() retrieved from the Vegan package and ordinate() in the Phyloseq package. Permutational multivariate analysis of variance (PERMANOVA) was conducted using the adonis() from the Vegan package (v2.5-3) [27]. Variance partitioning and significances for experimental factors were performed by running Vegan’s permutest() using 99,999 permutations. The same Bray–Curtis dissimilarity matrix was also used to make a neighbor-joining tree of microbiota in rice accessions. The Mantel test was conducted to find a correlation between the genetic distances of *Oryza* spp. and the compositional dissimilarity of microbiota. Additionally, cophylogenetic trees were constructed using the cophylo() in phytools package [29]. In order to quantify the topological congruencies between rice phylogeny and bacterial and fungal dendrograms, the Robinson-Foulds metric was used. The Robinson-Foulds scores and *p* values were calculated with the function RFmeasure() from the R script made available by Mazel and colleagues [30] based on 100,000 randomized trees. Taxa above relative abundance of 0.5% were visualized with the R package ggplot2 [31] for taxonomic composition analysis. Core OTUs were identified for wild and domesticated rice, respectively. The prevalence threshold for core OTUs was 95% (bacteria)/80% (fungi). A zero-inflated Gaussian distribution mixture model was used by applying fitZig() from metagenomeSeq. Moderated *t* tests were applied between wild and domesticated rice using the makeContrasts and eBayes commands retrieved from the R package Limma (v.3.34.9) [32]. Differences in the abundance were considered significant when FDR adjusted *p* values were lower than 0.01. Differentially abundant bacterial and fungal OTUs were visualized in Volcano plots with ggplot2. Tax4Fun2 (v1.0) was implemented in default settings to predict and compare the habitat-specific functional pathways and functional redundancy of bacterial communities of wild and domesticated rice from the partial region of 16S rRNA genes [33]. Ecological guilds of fungal OTUs were predicted using FUNGuild (v1.1) [34].

### Hierarchical clustering of OTUs

OTU abundance profiles were visualized in a hierarchically clustered dendrogram to detect the pattern difference of microbial compositions between wild and domesticated rice. OTUs that had more than 200 reads were used. The filtered OTU table was CSS-normalized/log-transformed. The OTU tables of bacteria and fungi were merged and uploaded to the Morpheus web site (https://software.broadinstitute.org/morpheus) for hierarchical clustering and visualization. Average linkage during hierarchical clustering both on OTUs (rows) and rice accessions (columns) based on the distance calculated with Spearman correlations were used.

### Generation of a classification model

The classification model was built by setting wild and domesticated rice (0 and 1, respectively) as a function of microbiota abundance. Two-thirds of the total samples were randomly sampled as the training set. ROC curves (ROCR package, v1.0.7) and ten-fold cross validation (caret package, v6.0-81) [35] were analyzed with the remaining test set in order to test which machine learning algorithm had the best performance among five classification methods in R: random forest (randomForest package, v. 4.6-14) [36], Support vector machine (SVM) (e1071 package, v1.7-0) [37], Naïve Bayes (e1071 package) [37], k-nearest neighbors (class package, v7.3-14) [38], and logistic regression (stats package, v3.4-4) [24]. The random forest (RF) classifier gave the highest area under ROC (AUC) score of 0.924 (bacteria)/0.889 (fungi) (Additional file 1: Figure S20) and highest cross-validation accuracy of 0.894 (bacteria) and 0.964 (fungi) compared to four other machine learning algorithms (Additional file 1: Table S4). Based on the results, RF classifier was chosen for further analyses. OTUs were ranked by their importance in contributing to the accuracy of wild/domesticated rice prediction in the RF model by calculating the mean decrease in Gini coefficient. This step was done using the importance() command in the randomForest R package. Ten-fold cross validations were performed while excluding less important OTUs to evaluate model performance as a function of inclusion of the top wild/domesticated rice discriminating OTUs using the rfcv() in the randomForest R package [39]. The minimum number of OTUs with the prediction error rate which is as low as the error rate of the full RF model 364(bacteria)/356(fungi) was determined. There was a rapid increase in the prediction error rate when the model included approximately less than 20 of the most important OTUs prompted the setting of the threshold to 20 (Additional file 1: Figure S21). The top 20 OTUs from the RF model of each kingdom were further categorized as wild-enriched, domesticated-enriched, or non-differential OTUs depending on the result of differential abundance test above.

### Microbial correlation networks

Networks were individually constructed to infer hub and complex associations between OTUs, for (1) wild rice, (2) domesticated rice, and (3) both wild and domesticated rice. In order to construct the co-occurrence networks, total 483 (256 bacterial and 227 fungal OTUs) and 415 OTUs (215 bacterial and 200 fungal OTUs) were used in wild and domesticated rice seeds, respectively. For the co-occurrence network of all seed samples, total 720 OTUs (364 bacterial OTUs and 356 fungal OTUs) were used. Multi-kingdom OTU tables (bacterial and fungal OTU tables merged) were used as an input for SparCC [40]. The SparCC analysis was conducted with compositionality-robust correlations from the median of 20 iterations and 100 bootstrap samples were used to infer pseudo *p* values. The inferred correlations were restricted to those having correlations > 0.3 or < − 0.3 (*p* < 0.05, two-sided) [41]. Visualization was done with Gephi (v0.9.2) [42], using the ForceAtlas2 layout. Within the networks, proportions of inter- and intra-kingdom edges were calculated and displayed into bar graphs [43]. Intra-kingdom refers to edges within bacterial or fungal OTUs, whereas inter-kingdom refers to edges between bacterial and fungal OTUs. To compare wild and domesticated rice networks, degree, betweenness centrality, closeness centrality, and eigenvector centrality were estimated using Gephi (v0.9.2). Hub OTUs of each network were defined as OTUs belonging to top 2% of degree and betweenness centrality. For wild rice, OTUs with degree greater than 12.8 and betweenness centrality higher than 0.090675 were defined as hub OTUs. For domesticated rice, OTUs with degree greater than 8.44 and betweenness centrality higher than 0.08536152 were selected as hub OTUs. For all rice network, OTUs showing degree greater than 6 and betweenness centrality higher than 0.05460252 were chosen as hub OTUs.

### Microbial vertical transmission analysis

Each cultivar was classified by breeding chronicles to decipher the vertical transmission of microbiotas. The pedigree information of each rice cultivar was obtained from Nongsaro (http://www.nongsaro.go.kr) at the Rural Development Administration, Korea. The pedigree was visualized using the Helium program (Additional file 13) [44]. In 25 cultivars, two breeding lines originating from Palgoeng and Dongjin were used. Six orphan cultivars were also included, which were not connected to any breeding lines. The distances in intra-breeding lines and inter-breeding lines were compared (Additional file 1: Figure S24a). Intra-breeding lines are a cross of an ancestor and one of their descendant cultivars. Inter-breeding lines consist of an ancestor cultivar and a descendant cultivar of other pedigrees. For further analysis, specific subsets which were linked by maternal inheritance were also used (Fig. 6a). Direct breeding lines and vertical breeding lines consist of direct (first-degree connection) and indirect mother-progeny connections, respectively (Milyang 95-Dongjinchal and Hwayeong-Sobi; Palgoeng-Shinseonchal and Palgoeng-Tamjin). Kin breeding lines consist of descendent cultivars which share three maternal ancestors (Shinseonchal-Tamjin, Daean-Koshihikari, and Tongil-Kinuhikari). Bray–Curtis distances of each component group were analyzed. To compare specified small groups, two direct breeding lines and a combined comparison with two vertical groups including one kin group were selected. OTUs in each component were compared by their presence or absence.

## Results

### Taxonomic structure and patterns of the rice seed microbiota

To elucidate the structure and community-driving forces of rice seed microbiota, bacterial and fungal communities from surface-sterilized seeds of 43 rice accessions (17 wild and 26 domesticated rice) were analyzed (Fig. 1; Additional file 2). The V4 regions of the 16S rRNA genes of bacteria and ITS2 regions of fungi were sequenced, generating total 16,268,117 reads on the Illumina Miseq platform (Additional file 3). To inhibit the amplification of plant mitochondrial and plastid DNAs, PCR blockers were used (Additional file 1: Figure S2 and Table S3; Additional file 5). After eliminating remaining plant DNAs, chimeras, and low-abundance operational taxonomic units (OTUs) with less than five reads across all the samples, 364 bacterial and 356 fungal OTUs were identified at 97% sequence similarity (Additional file 3).

Taxonomic classification of the bacterial sequences at the phylum level revealed a prevalence of a single phylum *Proteobacteria* (83.8%: *Gammaproteobacteria*, 60.1%; *Alphaproteobacteria*, 12.5%) (Additional file 1: Figure S3). Fungal reads were also monopolized by a single phylum *Ascomycota* (84.9%: *Dothideomycetes*, 58.1%; *Sordariomycetes*, 26.1%; *Eurotiomycetes*, 0.7%) (Additional file 1: Figure S5). When observed at the genus level, *Pantoea* (42.5%), *Methlyobacterium* (11.8%), *Curtobacterium* (9.3%), *Pseudomonas* (8.7%), and *Sphingomonas* (8.6%) dominated the total bacterial reads (Additional file 1: Figure S4). For fungi, *Curvularia* (23%), *Moesziomyces* (13.6%), *Fusarium* (7.8%), *Sacrocladium* (7.13%), and *Bipolaris* (7%) dominated the total reads (Additional file 1: Figure S6). Both bacterial and fungal genera showed significant differences among 43 *Oryza* accessions (Additional file 1: Figures S7-S12). In particular, fungal genera *Curvularia* and *Moesziomyces* showed dramatic differences between wild and domesticated rice (Additional file 1: Figures S8 and S10). Based on these results among the 43 rice accessions, it was further examined whether the speciation of the *Oryza* genus, domestication, or both might affect the variations of seed microbial communities.

### The speciation of the *Oryza* genus explains the variation of the seed microbiota

**Fig. 2:**
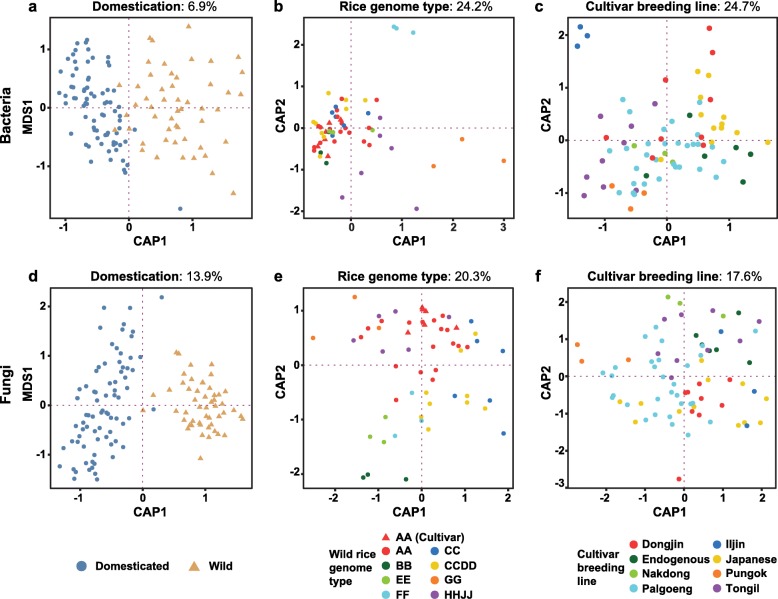
Constrained analysis of seed bacterial and fungal communities’ principal coordinates. **a**–**c** Variation of bacterial communities constrained by **a** domestication status, **b** genome group, and **c** breeding line. **d**–**f** Variation of fungal communities constrained by **d** domestication status, **e** genome group, and **f** breeding line. Cumulative sum scaling (CSS)/log transformed reads were used to calculate Bray–Curtis distances. Full datasets were used to investigate the variation by domestication. Wild and domesticated rice categorization explained 6.9% (bacteria) and 13.9% (fungi) of the overall variance (PERMANOVA, *p* < 0.001). To analyze the effects of genome groups and breeding lines, the subset consisting of 17 wild *Oryza* accessions and two varieties of *O. sativa* (Tongil and Nipponbare) and the subset of 25 varieties were used, respectively. Rice genome group explained 24.2% (bacteria) and 20.3% (fungi) of the total variance in the subset (PERMANOVA, *p* < 0.001). The breeding line explained 24.7% and 17.6%, bacteria and fungi each, of the variability of seed microbiome in 25 cultivars (PERMANOVA, *p* < 0.001). All CAP analyses were significant (Permutest, *p* < 1e-5)

To unveil shaping factors on seed microbiota, we first investigated the impact of the host speciation on the microbial structure. Considering that polyploidization is a prominent process in the evolution of higher plants, the genome group could have broader effects on the microbiota [45]. As rice genome AA group was overrepresented, a subset of 17 wild rice and two cultivars (Nipponbare and Tongil) were selected. In this subset, the rice genome group explained 24.2% (bacteria) and 20.3% (fungi) of the total variance (PERMANOVA, *p* < 0.001; Additional file 6). Consistent with these results, the CAP analysis revealed a significant separation among rice genome groups for both bacterial and fungal communities (Fig. 2b, e; Permutest, *p* < 1e-5). This ordination suggests a significant difference between the effects of genome group on bacterial and fungal communities.

Accordingly, it was tested whether the phylogenetic relationship of rice correlates with the dissimilarity distance of seed microbial communities. Cophylogenetic trees were constructed based on complete chloroplast sequences of rice accessions and Bray–Curtis dissimilarity matrix of the microbial communities (Additional file 1: Figure S13). In terms of phylosymbiosis, if effects of host genetic factors overwhelm those of other shaping factors, the topology of the rice phylogeny and the dendrogram of microbiota will be similar. Robinson-Foulds scores showed that the dendrogram of bacterial community had significant congruence with the phylogeny of *Oryza* spp. (Additional file 1: Figure S13a; RF = 0.857, *p* = 0.0016), whereas fungal community did not (Additional file 1: Figure S13b; RF = 0.971, *p* = 0.1776). The Mantel test again corroborated these results (Additional file 7). Taken together, the bacterial community seems to show greater correlation with rice speciation than the fungal one.

### Structure of seed microbial communities is shaped by domestication

To elucidate the effects of rice domestication on the microbiota, a coordinate analysis was performed on 43 accessions grouped into wild and domesticated rice. Domestication status explained 6.9% of the total variability in bacteria and 13.9% in fungi (PERMANOVA, *p* < 0.001; Additional file 6). The ordination of the CAP analysis also revealed a significant separation between seed microbial communities of wild and domesticated rice for both bacteria and fungi (Fig. 2a, d; Permutest, *p* < 1e-5). Notably, the fungal community seems to be more influenced by domestication than the bacterial community is. This separation was also found in an unconstrained condition (Additional file 1: Figure S14). These results indicate that the compositional variations of bacterial and fungal communities could be more related to domestication, although other processes were also significant for the compositional variations of microbial communities (Fig. 2 and Additional file 6).

However, given that seeds were acquired from two different locations, the Philippines and South Korea, there may exist the possibility that geographical locations contribute to the observed differences. To quantify the effects of geographical locations, we performed the PERMANOVA. There was no significant effect of geographical location in the bacterial communities (*R*^2^ = 0.01, *p* = 0.0662). Although geographical location contributed to the variations in the fungal communities (*R*^2^ = 0.01811, *p* = 0.001), the effect size was much lower than domestication (*R*^2^ = 0.13883, *p* = 0.001). We also analyzed the microbial communities of *O*. *nivara* (wild rice) obtained from Korea to further validate the effects of geographic location or domestication. Bacterial community of *O*. *nivara* was closer to *O*. *sativa* Akibare, but there was no distinct clustering pattern throughout all rice accessions along with geographical location or domestication (Additional file 1: Figure S15). However, fungal community of *O*. *nivara* was closer to wild rice accessions despite the difference in the geographical location (Additional file 1: Figure S15). Based on these results, domestication was considered as the major effect on the variations in seed bacterial and fungal communities despite the geographical difference.

### Domestication increases dominance of fewer bacterial and fungal OTUs

To identify domestication effects on the diversity of seed microbial community, we investigated the alpha diversity of microbial communities between wild and domesticated rice (Additional file 1: Figures S16, S17, and S18). For the bacterial community, domesticated rice had higher observed OTUs than wild rice (Wilcoxon rank-sum test, *p* = 0.01592; Additional file 1: Figure S17). However, for fungal community, wild rice had higher alpha diversity than domesticated rice (Wilcoxon rank-sum test, *p* < 0.01; Additional file 1: Figure S17). We further investigated how domestication affects the abundance distribution of OTUs within each community. Bacterial and fungal communities of domesticated rice had a higher abundance inequality than those of the wild rice (Additional file 1: Figure S16b, e). Further, 10.2% (bacteria) and 12% (fungi) of the OTUs accounted for 80% of the total abundance in the domesticated rice, whereas 14.8% (bacteria) and 14.5% (fungi) of the OTUs accounted for the 80% of the total abundance in the wild rice (Additional file 1: Figure S16c, f). These results suggest that a few OTUs have dominated the vast majority of the total abundance. The phenomenon of domination by few OTUs, or the manifestation of the Pareto rule (80-20 rule) in seed microbial community, raised a question on how entire OTUs are affected and which OTUs are affected by domestication.

### Random forest modeling identifies domestication-associated bacterial and fungal OTUs

**Fig. 3:**
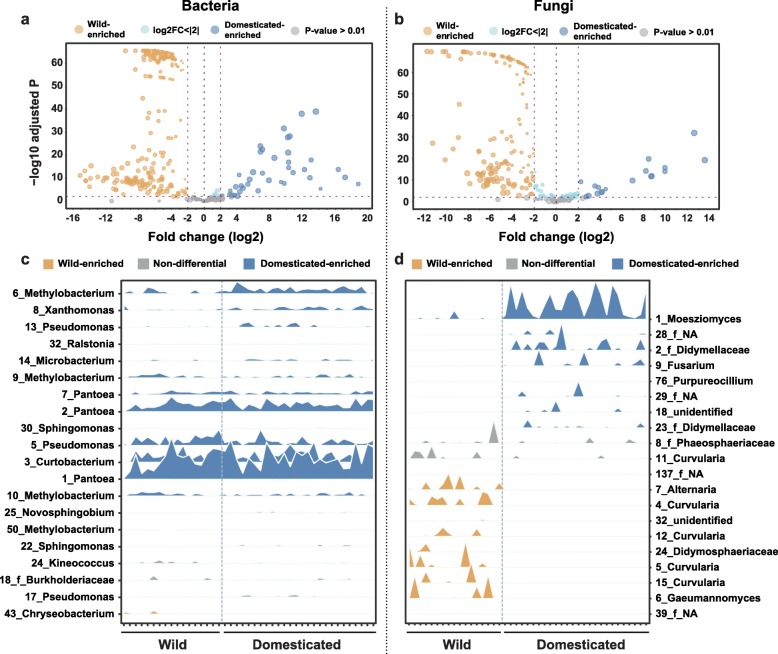
OTUs responsible for the differences during domestication of rice are revealed by a differential abundance test and random forest classification. **a**, **b** Volcano plot visualizing differentially abundant **a** bacterial and **b** fungal OTUs between the 17 wild and 26 domesticated rice accessions. The comparison was made using a zero-inflated Gaussian distribution mixture model on the CSS normalized OTU tables followed by a moderated *t* test and a Bayesian approach. Data from all three replicates of each accession were used. This test revealed 39 bacterial and 18 fungal OTUs that were significantly enriched in domesticated rice (above four folds (log2 Fold change > 2), FDR < 0.01). A total of 224 bacterial and 167 fungal OTUs were enriched in wild rice (above 4 folds (log2 Fold change < − 2), FDR < 0.01). **c**, **d** Relative abundance profiles for the Top 20 operational taxonomic units (OTUs) best discriminating wild and domesticated rice accessions with Random forest (RF) classifier in c bacterial and d fungal communities. OTUs are colored by their categorization as ‘wild-enriched,’ ‘domesticated-enriched,’ and ‘non-differential’ according to the result of a differential abundance test in **a** and **b**. The RF model with top 20 OTUs were constructed with a 10-fold cross validation method. OTUs were ranked by their importance in contributing to the accuracy of wild and domesticated rice prediction in the RF model by calculating the mean decrease in Gini coefficient. OTUs are ordered along the *y*-axis by rank of importance within each of the three categories

To detect domestication-related patterns of OTUs, we constructed a hierarchically clustered heat map of OTUs over 200 reads across all samples (Additional file 1: Figure S19; Additional file 8). We observed eight clusters consisting of bacterial and fungal OTUs (seven clusters abundant in domesticated rice and one cluster abundant in wild rice). This analysis suggests the effects of rice domestication on the microbiota composition; thus, we further investigated these effects. Through the differential abundance test between wild and domesticated rice, we obtained a total of 263 bacterial (224 wild-enriched OTUs; 39 domesticated-enriched OTUs) and 185 fungal OTUs (167 wild-enriched OTUs; 18 domesticated-enriched OTUs) potentially affected by domestication (|log2 Fold change| > 2, FDR < 0.01) (Fig. 3a, b; Additional files 9 and 10). This asymmetric pattern could be related to the loss of microbial diversity during domestication.

To zero in on the ‘most important’ OTUs associated with domestication, we modeled ‘wild’ and ‘domesticated’ categories (dependent variable) as a function of OTU reads (independent variable). Using the RF model, the top 20 OTUs were chosen as they almost had the same cross-validation error rate as the RF model with all 364 bacterial or 356 fungal OTUs (Additional file 1: Figure S21). Top 20 bacterial OTUs consisted of *Proteobacteria* (16 OTUs), *Actinobacteria* (three OTUs), and *Bacteroidetes* (one OTU). Among bacterial OTUs showing significant differences in the abundance distribution, most bacterial OTUs were ‘Domesticated-enriched’ except one OTU (*Bacteroidetes*; *Chryseobacterium*) (Fig. 3c). This result suggests that the enrichment of OTUs belonging to *Proteobacteria* and *Actinobacteria* is associated with rice domestication. In the case of fungal community, the top 20 discriminant OTUs were composed of *Ascomycota* (18 OTUs) and *Basidiomycota* (two OTUs) (Fig. 3d). The significantly differentially distributed OTUs showed the opposite enrichment patterns according to the domestication status. These results suggest that the switching of dominant genera could be the major response of the fungal community to domestication, consistent with the observation of their relative abundance (Additional file 1: Figure S10).

### Co-occurrence patterns of microbial communities between wild and domesticated rice

**Fig. 4:**
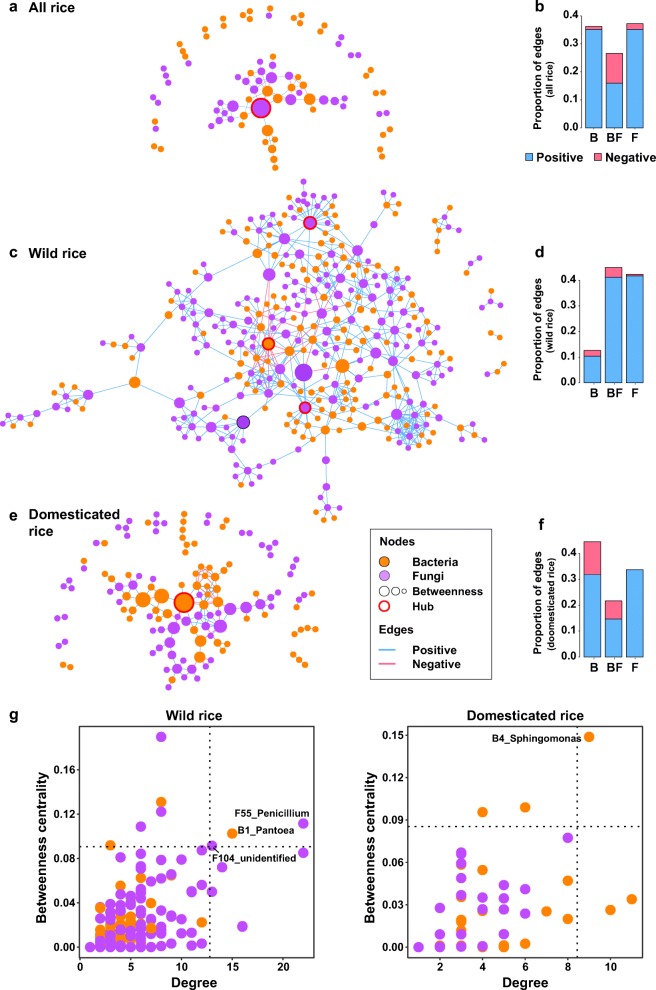
Microbial networks of the rice seed endosphere microbiotas. **a**, **c**, **e** Co-occurrence-based network of seed microbial OTUs detected in **a** all wild/domesticated rice accessions, **c** wild rice accessions, and **e** domesticated rice accessions. Each node corresponds to an OTU, and edges between nodes correspond to either positive (light blue) or negative (pink) correlations inferred from OTU abundance profiles using the SparCC method (pseudo *p* < 0.05, correlation values < − 0.3 or > 0.3). OTUs belonging to different microbial kingdoms have different color codes (bacteria, orange; fungi, purple), and node size reflects their betweenness centrality in the seed endosphere. **b**, **d**, **f** Bar graph shows the proportion of inter- and intra-kingdom edges of positive (light blue) or negative (pink) correlations in the seed endosphere network. B, bacteria intra-kingdom; F, fungi intra-kingdom; BF, bacteria-fungi interkingdom association. **g** Hub OTUs of wild (left panel) and domesticated rice seeds (right panel). Dashed lines indicate the threshold estimated by the values which top 2% of nodes showed

The differences in the response of bacterial and fungal communities to the domestication suggest that overall co-occurrence patterns of OTUs in wild and domesticated rice would be different from each other. To examine the variations in the microbial network structure by domestication, we built co-occurrence networks of both bacteria and fungi in (1) all rice accessions (Fig. 4a), (2) within only wild rice (Fig. 4c), and (3) within only domesticated rice (Fig. 4e). Co-occurrence network of wild rice consisted of 361 nodes and 673 edges, whereas those of domesticated rice consisted of 129 nodes and 159 edges. The discrepancy in the total numbers of OTUs in the network input data and that of network nodes suggest tighter associations among OTUs in wild rice than in domesticated rice. On the other hand, the numbers of nodes and edges decreased to 82 nodes and 105 edges in the all rice network. This indicates the compositional gap between wild and domesticated rice, resulting in low correlation coefficients among OTUs. This discrepancy between microbial communities of wild and domesticated rice was also identified in network centrality indices (Additional file 1: Figure S22). In particular, higher degree and betweenness centrality in the network of wild rice indicate that the connectivity among nodes is higher in wild rice.

We further identified hub OTUs (OTUs showing high degree and betweenness centrality) to investigate how the variations in microbial community affect network hubs. In the microbial network of domesticated rice, the only one hub OTUs were identified, bacterial OTUs belonging to *Sphingomonas* (B4_Sphingomonas) (Fig. 4g; Additional file 11). On the other hand, one bacterial OTU (B1_Pantoea) and two fungal OTUs (F55_Penicillium and F104_unidentified) were found as the hubs of the network of wild rice (Fig. 4g). By comparing the hubs in the microbial networks of wild and domesticated rice, it was evident that the hubs of the microbial network in domesticated rice were bacteria, whereas associations within the wild rice microbial network were mostly centered around fungal hubs. Furthermore, network centrality indices between bacterial and fungal nodes also showed fungi were more important in the microbial network of wild rice seed than in the domesticated one (Additional file 1: Figure S23).

### Evidence for vertical transmission of seed microbiota

**Fig. 5:**
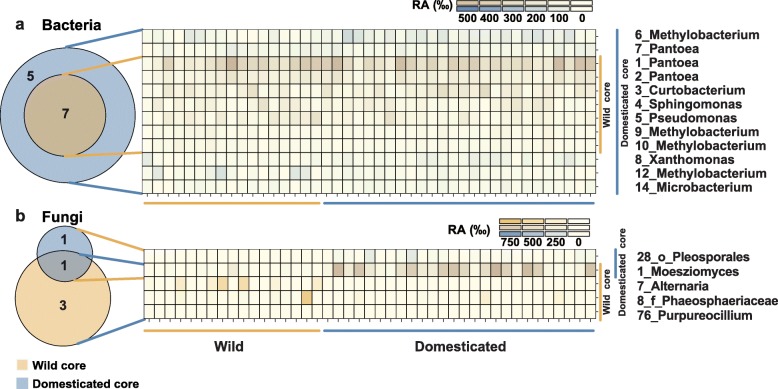
Venn diagram and abundance profile heatmaps of core bacterial and fungal OTUs. **a**, **b** Core OTUs of **a** bacterial and **b** fungal communities of 43 genotypes. Core OTUs were calculated respectively for the domesticated rice group and the wild rice group. The prevalence threshold for core OTUs was 95% (bacteria) and 80% (fungi) out of rice accessions. Heatmaps indicate the relative abundance of OTUs in rice accessions. RA, relative abundance.

The existence of the all rice network suggests the conserved microbial communities between wild and domesticated rice during rice evolution and domestication (Fig. 4a). In order to find the conserved fraction, we identified the core OTUs of wild and domesticated rice which were present in more than 95% (bacteria)/80% (fungi) in all rice accessions (Fig. 5). In bacteria, 12 core OTUs of domesticated rice included all seven core OTUs of wild rice (Fig. 5a; Additional file 12). In the fungal community, four core OTUs of the wild rice and two core OTUs of domesticated rice were identified (Fig. 5b; Additional file 12). Only one OTU overlapped between the two groups and it was assigned to *Moesziomyces*. Taken together, the bacterial biota seems more conserved and less variable than the fungal biota during domestication.

Core OTUs suggest the existence of the possible way to inherit or conserve microbial communities across generations. To prove this, we investigated the transmission mechanism of microbiota from generation to generation in domesticated rice. Based on a pedigree of Korean rice cultivars constructed by tracking the origin and cross-breeding history (Additional file 13), the tested cultivars were grouped into eight breeding lines (Fig. 1). Bray–Curtis dissimilarity metrics in two breeding lines with an orphan group were applied with binary estimation [46] (Additional file 1: Figure S24a). In the bacterial community of the Palgoeng breeding line, the dissimilarity of the intra-breeding line was lower than that of the inter-breeding line. The distance within the orphan breeding line was the most dissimilar compared to the other breeding lines (Additional file 1: Figure S24b). Although less distinctive than the bacterial community, the distribution of the fungal community’s median in Palgoeng showed a similar pattern (Additional file 1: Figure S24c). These results suggest that some fraction of the microbiota is shared or inherited in the specific breeding lines.

**Fig. 6:**
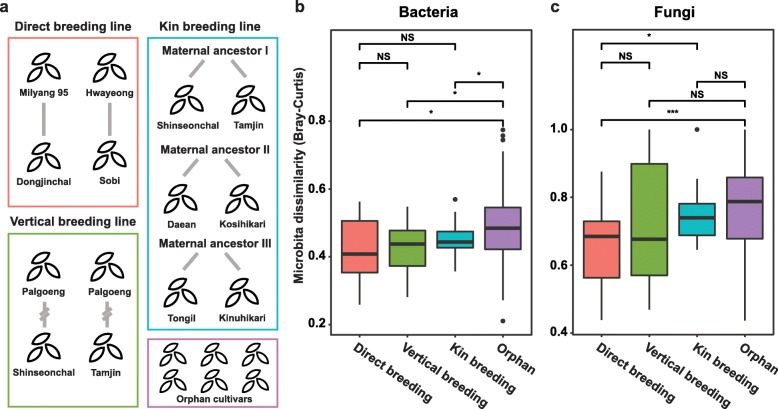
Vertical transmission analysis on cultivar seed microbiome. **a** Compares four groupings: direct, vertical, kin breeding line, and orphan cultivars. All breeding linkages were based on maternal relationship. Two sets of cultivars were linked by direct mother-progeny connection (Milyang 95-Dongjinchal and Hwayeong-Sobi). Two sets had common breeding generations of ancestor-descendant linkage as siblings (Palgoeng–Shinseonchal and Palgoeng–Tamjin). Each pair of cultivars in kin breeding line shared common maternal ancestor (Shinseonchal-Tamjin, Daean- Koshihikari, and Tongil-Kinuhikari). Orphan domesticated cultivars were not found to connected to other cultivars in this study (Pungok, Jodongji, Joshinryeok, Akibare, Nakdong and Iljin). **b**, **c** Dissimilarity distance calculated in Bray-Curtis distance of CSS normalized OTU table within each group **b** bacterial and **c** fungal communities. Statistically significant differences between dissimilarity distances were determined by Wilcoxon rank-sum test (***‚ *p* < 0.001; **‚ *p* < 0.01; *‚ *p* <0.05; NS, *p* > 0.05)

To further understand the mechanism underlying the vertical transmission of microbiota, we also analyzed the composition of microbiota from selected cultivars that were maternally connected (Fig. 6a). Dissimilarity is the lowest in direct breeding lines followed by vertical and kin breeding lines. The orphan group showed the highest dissimilarity among all group comparisons (Fig. 6b). Fungal taxa showed a similar pattern to bacteria (Fig. 6c). When analyzed at the OTU level, two cultivars shared almost half of the total OTUs that were shared among all cultivars in direct, vertical, and kin breeding lines (Additional file 1: Figure S25). These findings suggest that each maternal connection shares not only core OTUs but also connection-specific OTUs. This strongly indicates that maternal transmission would be a major path of vertical transmission of microbiota in rice seeds.

## Discussion

The close associations between hosts and their microbiomes suggest a holobiont concept. The holobiont concept considers a holobiont with its hologenome as a distinct biological and evolutionary entity on which natural selection operates [47]. In this context, host speciation can directly shape structures of microbiotas in terms of phylosymbiosis. Significant phylosymbiosis demonstrates that microbial communities are deterministically assembled by host factors [48]. Bacterial communities of animals showed significant phylosymbiosis in a controlled environment [49] and in animals which share similar diets [50]. We demonstrated that seed bacterial community showed a weak but significant phylosymbiosis (Additional file 1: Figure S13a; Additional file 7). We also demonstrated that host genome type significantly affects compositional variations in bacterial communities (Fig. 2b; Additional file 6). These results suggest that the eco-evolutionary relationships in bacterial community can be derived from genomic differences. In particular, the topological congruency shown in early divergent *Oryza* spp. suggests that bacterial community may undergo a deterministic assembly via host factors of GG, FF, and HHJJ genome types. On the other hand, the fungal community did not show a significant phylosymbiotic relationship (Additional file 1: Figure S13b; Additional file 7). This suggests that the assembly of fungal communities may be a stochastic process governed by external factors rather than by host factors.

The effects of geographical locations have been reported in microbial communities residing in rhizosphere/root endosphere [5] and phyllosphere [51]. However, which factors shape seed microbial communities are controversial. Geographical signatures are major shaping factors of bacterial communities of grape [52]. However, bacterial communities associated with surface-sterilized seeds of maize, rice, and pumpkin were more affected by plant genotypes than geographical factors [53–56]. We found that the effect of domestication exceeded that of geographical locations in variations of seed microbial communities. Our findings and previous reports suggest that seed endosphere is relatively more stable than other parts of plants against environmental changes.

A momentous finding is that bacterial and fungal communities differently responded to domestication. In the case of bacteria, the number of total and core OTUs and their abundances were increased following domestication (Fig. 5a; Additional file 1: Figure S17). In addition, RF modeling revealed that one major impact of domestication is the enrichment of abundance of dominant bacterial genera (Fig. 3c). The change in fungal membership is also a key response to domestication (Fig. 3d). In particular, we found that bacterial OTUs enriched in domesticated rice seeds belonged to *Proteobacteria* and *Actinobacteria*, whereas OTUs belonging to *Bacteroidetes* were enriched in seed bacterial community of wild rice. These distribution patterns of bacterial phyla in wild and domesticated plants were also reported in rhizosphere bacterial communities associated with sugar beet, *Arabidopsis*, barley, and lettuce [57]. These findings indicate that plant domestication affects similar shifts in the bacterial communities of various plants and tissues at higher taxonomic level. Similar enrichment patterns between seed and rhizosphere suggest that host factors may be involved in the assembly of seed and rhizosphere microbial communities.

Domestication also affected the structure of microbial co-occurrence networks. In wild rice, the network hubs consisted of both bacterial and fungal OTUs, whereas the hub was exclusively identified as bacterial OTU in domesticated rice. Considering that crop domestication is a process driven by loss-of-function alleles [1] and indels in plant genomes [58, 59], domesticated rice might have lost the genes responsible for symbiotic/mutualistic relationships with fungal species. Domestication also affected the overall network structures of wild and domesticated rice seeds. The number of edges (putative associations) decreased in the network of domesticated rice compared to that of wild rice (Fig. 4a–f). Network connectivity was also higher in wild rice (Additional file 1: Figure S22). The differences in network complexity between wild and domesticated rice may be related to environmental effects as the consequences of domestication. Domestication accompanied the changes in not only genetic properties of crop plants but also in environmental conditions which surround microbial communities [60]. The changes in environmental conditions are caused by management practices which are applied for supporting the physiological activities of domesticated crop plants. Management practices including fertilization could change nutrient availability, resulting in altering microbial composition, functions, and microbial associations. This is further supported by the network complexity of wheat root fungal community which decreased according to increase in the degree of agricultural intensification [61]. Although the interpretation on predicted community functions from partial marker genes is cautious, the predicted functional profiles of bacterial and fungal communities suggest putative link between changes in agronomic traits and microbial functions in seeds (Additional file 1: Supporting information). The findings suggest that both host genetic and abiotic factors which are changed during domestication might shape seed microbial communities.

Another key finding is that seed microbiota can be vertically inherited. The microbiotas of human and mammalians are known to be vertically transmitted and be reshaped by environmental influences over time [46, 62]. In particular, gut microbiotas of humans and animals are easily affected by external factors such as diet [63], which could make it harder to investigate the long-term inheritance of microbiotas. However, the seed microbiota gave us the opportunity to identify a conserved fraction of microbiota. We found half of bacterial OTUs associated with domesticated rice were shared in both mother and progeny varieties in specific breeding lines. Fungal community was also shared but to a lesser degree than the bacterial community (Fig. 6c; Additional file 1: Figure S24c). These putatively inherited fractions included core OTUs, especially in bacteria, suggesting that core bacterial community may have mutualistic relationships with the host. Above all, our findings suggest that seed microbiota may be inherited from mother to progeny. One of the possible underlying mechanisms is transmission via the shoot apical meristem (SAM). The SAM can be differentiated into other tissues including reproductive organs which are developed as seeds. Microbes inhabiting the SAM may move upward following the development of SAM as a ‘meristematic escalator’ or ‘meristematic highway.’ The colonization of bacteria in shoot meristem suggests that the movement of microbes via meristematic tissues is a possible way that seeds are colonized [64].

## Conclusion

We evaluated the effects of both host speciation and domestication as long- and short-term evolutionary factors on seed microbiota using 43 wild and domesticated rice accessions. Experimentally, our approach vastly improved the resolution of the seed bacterial community, which previous approaches missed. In particular, a parallel investigation on the fungal community unveiled hidden responses of the seed microbiota to host evolution and the complex inter-kingdom associations with the bacterial community. In conclusion, the speciation in early divergent rice deterministically affected bacterial community. However, the host speciation stochastically shapes both bacterial and fungal communities. On the other hand, domestication contributed to community assembly in a more deterministic way by dramatic changes in host and environmental factors. Our study also provides integrated evolutionary insights on seed microbiome, the starting point of the holobiome of plants, microbial communities, and surrounding environments.

## Supplementary information

**Additional file 1: Figure S1.** Abundance profiles depending on different normalization and transformation methods. The frequency distribution of OTUs was plotted to compare the effects of normalization methods. CSS normalization with and without log2(1+x) transformation, rarefying by 1,000 reads, relative abundance, and Hellinger transformation (square root of relative abundance) methods were tested. CSS normalization with log2(1+x) transformation and Hellinger transformation had distributions close to Gaussian distribution. CSS, cumulative sum scaling. **Figure S2.** The use of Peptide Nucleic Acid (PNA) clamps during the PCR step reduced mitochondrial and plastid DNA contamination from the rice plant. (a) Usable reads in raw sequence reads of all sample replicates. ‘Usable reads’ (light blue) are reads that excluded Chloroplast (light green) and Mitochondrial (orange) reads from the raw reads. With the use of PNA clamps more than about 10,000 reads were usable per sample replicate, whereas without the use of PNA clamps much less reads were usable. We compared the resolution of taxonomic identification in relative abundance (RA) bar plots at the (b) family level and (c) genus level of bacterial taxa of 43 rice accessions’ seeds. High abundance taxonomic groups did not differ between with and without PNA clamps, but low abundance taxonomic groups were more identified in the PNA clamp-used samples at both the family and genus level. Taxonomic groups with less than 5 ‰ (per-mille) of each samples were labeled as ‘Low abundance’. Each technical replicate comprised a pool of three sets of three grains. Further statistical information on average reads, OTUs and Shannon diversity index, is detailed in Additional file 5. Abbreviations for rice accessions are available in **Table S1**. **Figure S3.** Relative abundance (RA) in the (a) phylum, (b) class and (c) order level of bacterial taxa in the seeds of 43 rice accessions. Low abundance taxonomic groups with less than 5 ‰ (per-mille) of each samples are highlighted in gray. Unidentified taxonomic groups are indicated in black. Each technical replicate comprised a pool of three sets of three grains. Abbreviations for rice accessions are available in **Table S1**. **Figure S4.** Relative abundance (RA) in the (a) family and (b) genus level of bacterial taxa in the seeds of 43 rice accessions. Low abundance taxonomic groups with less than 5 ‰ (per-mille) of each samples are highlighted in gray. Unidentified taxonomic groups are indicated in black. Each technical replicate comprised a pool of three sets of three grains. Abbreviations for rice accessions are available in **Table S1**. **Figure S5.** Relative abundance (RA) in the (a) phylum, (b) class, and (c) order level of fungal taxa in the seeds of 43 rice accessions. Low abundance taxonomic groups with less than 5 ‰ (per-mille) of each samples are highlighted in gray. Unidentified taxonomic groups are indicated in black. Each technical replicate comprised a pool of three sets of three grains. Abbreviations for rice accessions are available in **Table S1**. **Figure S6.** Relative abundance (RA) in the (a) family and (b) genus level of fungal taxa in the seeds of 43 rice accessions. Low abundance taxonomic groups with less than 5 ‰ (per-mille) of each samples are highlighted in gray. Unidentified taxonomic groups are indicated in black. Each technical replicate comprised a pool of three sets of three grains. Abbreviations for rice accessions are available in **Table S1**. **Figure S7.** Relative abundance of major genera of bacterial biota. Six major genera in bacterial biotas are selected based on the total sequence reads. Statistically significant differences between group means of relative abundance were determined by one-way ANOVA (P<0.05). Three replicates per an accession were used. Different letters indicate statistically significant differences. Abbreviations for rice accessions are available in **Table S1**. **Figure S8.** Relative abundance of major genera of fungal biota. Six major genera in fungal biota are selected based on the total sequence reads. Statistically significant differences between group means of relative abundance were determined by one-way ANOVA (P<0.05). Three replicates per accession were used. Different letters indicate statistically significant differences. Abbreviations for rice accessions are available in **Table S1**. **Figure S9.** Comparing relative abundance of major genera of bacteria between domesticated and wild rice accessions. Six major genera of bacterial biota are selected based on the total sequence reads in wild and domesticated rice accessions, respectively. Statistically significant differences between group means of relative abundance were determined by one-way ANOVA (P<0.05). Three replicates per accession were used. Different letters indicate statistically significant differences. Abbreviations for rice accessions are available in **Table S1**. **Figure S10.** Comparing relative abundance of major genera of fungi between domesticated and wild rice accessions. Six major genera in fungal biota are selected based on the total sequence reads in wild and domesticated rice accessions, respectively. Statistically significant differences between group means of relative abundance were determined by one-way ANOVA (P<0.05). Three replicates per accession were used. Different letters indicate statistically significant differences. Abbreviations for rice accessions are available in **Table S1**. **Figure S11.** Comparing relative abundance of minor genera of bacterial biota among rice accessions. Six minor genera in bacterial biota are selected based on the total sequence reads. Statistically significant differences between group means of relative abundance were determined by one-way ANOVA (P<0.05). Three replicates per accession were used. Different letters indicate statistically significant differences. Abbreviations for rice accessions are available in **Table S1**. **Figure S12.** Comparing relative abundance of minor genera of fungal biota among rice accessions. Six minor genera in fungal biota are selected based on the total sequence reads. Statistically significant differences between group means of relative abundance were determined by one-way ANOVA (P<0.05). Three replicates per accession were used. Different letters indicate statistically significant differences. Abbreviations for rice accessions are available in **Table S1**. **Figure S13.** Cophylogenetic tree of *Oryza* species and dendrogram of hierarchical clustering of Bray-Curtis distance of (a) bacterial and (b) fungal microbiotas. The phylogenetic tree of 17 *Oryza* spp. and 2 *O. sativa* cultivars (Tongil and Nipponbare). The tree was constructed based on the chloroplast genome sequence. RAxML program was used to draw Maximum Likelihood (ML) tree with 1,000 bootstraps. Dendrograms of bacterial and fungal biotas were constructed based on hierarchical clustering of Bray-Curtis distance using hclust() in R. Cophylogenetic trees were generated using the cophylo() in ‘phytools’ package. Robinson-Foulds score was calculated based on 100,000 randomized trees to quantify the topological congruency of host phylogeny and dendrograms of bacterial and fungal biotas. Bacterial biota showed significant eco-evolutionary relationships with rice phylogeny (RF = 0.857, P = 0.0016). **Figure S14.** Unconstrained principal coordinate ordination (PCoA) of the seed microbial communities. PCoA analysis of bacterial and fungal diversity in the seed endosphere was done with pairwise Bray-Curtis distances of all 43 rice accessions used in this study. CSS/log transformed reads were used to calculate Bray–Curtis distances. Each point represents each sample replicates and was colored by (a) wild and domesticated rice categorization and (b) rice genome type (AA, BB, CC, CCDD, EE, FF, GG, and HHJJ). **Figure S15.** Dendrogram of bacterial and fungal communities of 43 rice accessions based on Bray-Curtis distance. The dendrograms of bacterial and fungal communities were constructed using Bray-Curtis distance. Upper and lower panels indicate bacterial and fungal communities, respectively. The abbreviations of each accession are available in **Table S1**. **Figure S16.** Domestication effects on alpha diversity and community structure measured by cumulative abundance and percentage of OTUs. (a and d) Alpha diversity Shannon index comparing wild and domesticated rice seed (a) bacterial and (d) fungal communities. Wild rice includes 17 rice species and domesticated rice includes 25 *O*. *sativa* cultivars and *O*. *glaberrima*. (b and e) Cumulative abundance of total abundance (Y-axis) plotted against cumulative percentage of OTUs from the lowest to highest abundance (X-axis) in (b) bacterial and (e) fungal biotas. This graph (Lorenz-curve) was plotted to show how much the community abundance was dominated by few OTUs in wild and domesticated rice seed microbiome. The larger the area between the curve and the diagonal line, the higher the inequality. (c and f) Diagram show the percentage of total OTUs accounting for 80% of the total abundance (normalized sequence reads) in (c) bacterial and (f) fungal biotas. This diagram demonstrates the Pareto rule (80-20 principle) in the seed microbiome and compares wild rice to their domesticated counterpart. **Figure S17.** Alpha diversity comparison of wild and domesticated rice seed microbiota. Observed OTUs, inverse Simpson index, and Shannon index of 17 wild rice accessions and 26 domesticated rice accessions were calculated after Hellinger transformation of OTU tables. In bacteria, the number of observed OTUs was significantly higher in domesticated rice seeds than the wild counterpart (Wilcoxon rank-sum test, P < 0.05) but the differences in inverse Simpson and Shannon indices were not significant. In fungi, the number of observed OTUs, inverse Simpson and Shannon indices were significantly higher in wild rice seeds than the domesticated counterpart (Wilcoxon rank-sum test, P < 0.05). Wild, wild rice; Dom, domesticated rice. **Figure S18.** Alpha diversity comparison of all 43 rice accession seed bacterial and fungal biotas. The comparison of Shannon index between bacteria and fungi of each rice accessions shows that bacterial Shannon index (green) tends to be higher than fungal Shannon index (orange) in each rice seed microbial communities. Shannon index were calculated after Hellinger transformation of bacterial and fungal OTU tables. Abbreviations for rice accessions are available in **Table S1**. **Figure S19.** Heatmap shows a distinct pattern of wild and domesticated rice seed colonizers. Bacterial and fungal OTUs with more than 200 reads in the total sequence (127 bacterial OTUs, 108 fungal OTUs) were merged after CSS/log normalization of read counts. Samples and OTUs were hierarchically grouped (group-average linkage) based on the pairwise Spearman correlations both row-wise and column-wise. Hierarchical clustering and visualization was done on the Morpheus web platform (https://software.broadinstitute.org/morpheus). **Figure S20.** ROC curve and Out-of-bag error rate of full Random Forest (RF) model. Wild and domesticated rice category was set as dependent variables to be predicted with OTUs in 129 samples in 43 rice accessions (3 replicates). OTU tables were CSS/log normalized. Two-thirds of the samples of total samples were randomly sampled for the training set. (a) ROC curves were plotted with the remaining test set in order to test which machine learning algorithm had the best performance among 5 classifications: Support vector machine (SVM) Naïve Bayes, k-nearest neighbors, logistic regression. The number of trees for RF model was set to 1,500. Area under ROC curve (AUC) was calculated to measure 5 classifier’s performance and Random Forest model performed best (bacteria, AUC=0.924; fungi, AUC=0.889). (b) Out-of-bag error rate of RF models according to the number of trees was used. We used 1,500 trees which were in the range having the lowest Out-of-bag error rate. **Figure S21.** Cross-validation error of Random Forest (RF) model with differing numbers of OTUs to predict wild and domesticated rice categories. To evaluate model performance as a function of inclusion of the top wild/domesticated rice-discriminating OTUs, 10-fold cross validation was performed while gradually excluding less important OTUs. The prediction error rate was kept low from 364 bacterial and 356 fungal OTUs to approximately 20 OTUs. A rapid increase in the prediction error rate occurred when less than 20 of the most important OTUs were included. **Figure S22.** Comparison on network attributes between microbial co-occurrence networks of wild and domesticated rice. Statistically significant differences between dissimilarity distances were determined by Wilcoxon rank-sum test (****, *P* < 0.0001; ***, *P* < 0.001; **, *P* < 0.01; *, *P* < 0.05; ns, *P* > 0.05). The red dashed lines indicate the mean values of each centrality indices. **Figure S23.** Comparison on network attributes between bacterial and fungal nodes in microbial co-occurrence networks of wild and domesticated rice. Upper and lower panels indicate co-occurrence networks of wild and domesticated rice, respectively. The blue dashed lines indicate the mean values of each centrality. Statistically significant differences between dissimilarity distances were determined by Wilcoxon rank-sum test (****, P < 0.0001; ***, P < 0.001; **, P < 0.01; *, P < 0.05; ns, P > 0.05). **Figure S24.** Evidence of vertical transmission by comparing Bray-Curtis distances of Palgoeng and Dongjin breeding lines. (a) Diagram showing compared groups: Palgoeng and Dongjin breeding lines, and orphan cultivars. Dissimilarity distance was calculated with Bray-Curtis distance of CSS normalized OTU table within groups and between groups (Palgoeng inter and Dongjin inter) in (b) bacterial and (c) fungal communities. Statistically significant differences between dissimilarity distances were determined by Wilcoxon rank-sum test (***, P < 0.001; **, P < 0.01; *, P < 0.05; NS, P > 0.05). Three replicates per accession were used. Microbiota dissimilarity estimated within a breeding line (dissimilarity distance between an ancestor and their descendant cultivars) was indicated as ‘intra’. Microbiota dissimilarity estimated between breeding lines (dissimilarity distance between an ancestor and descendant cultivars of other pedigree) was indicated as ‘inter’. **Figure S25.** Bacterial and fungal OTUs commonly detected in separate lines. To compare taxonomical transmission tendency, we selected two first-degree lines (Milyang 95-Dongjinchal and Hwayeong-Sobi) and one common ancestor group (Palgoeng-Tamjin and Palgoeng-Shinseonchal). We showed shared OTUs in each component by presence/absence concept. The numbers in each circle mean the number of bacterial and fungal OTUs (Bacteria/Fungi). **Figure S26.** Distribution of ecological guilds of fungal communities associated with wild and domesticated rice seeds. (a) Distribution of fungal guilds in each rice accession. (b) Relative abundance of fungal guilds in the wild and domesticated rice. (c-f) Statistical analyses on the relative abundance of fungal guilds. (c) Putative plant pathogens; (d) Undefined saprotrophs; (e) Animal pathogen-Endophyte-Plant pathogen-Wood saprotroph; (f) Animal pathogen-Plant pathogen-Undefined saprotroph. The red dashed lines indicate the mean abundance of each guild. Asterisk indicates the significance based on unpaired Wilcoxon rank sum test. ****, p < 0.0001; ***, p < 0.001; **, p < 0.01; *, p < 0.05, ns (non-significant), p >0.05. **Table S1.** Abbreviation of rice accessions used for further analyses. **Table S2.** Primers used in this study. **Table S3**. PCR blockers used in this study. **Table S4**. Evaluation on accuracy of 5 classifier models.

**Additional file 2.** Rice accessions used in this study

**Additional file 3.** Eliminated sequence reads through filtering steps

**Additional file 4.** Filtered fungal OTUs annotated as rice sequences in all top 10 blastn hits

**Additional file 5.** Comparison of sequence read and number of bacterial OTUs detected with and without PCR blockers

**Additional file 6.** PERMANOVA analysis results

**Additional file 7.** Mantel test results for bacterial and fungal biotas

**Additional file 8.** Clusters of OTUs infered with hierarchical clustering

**Additional file 9.** Differentially abundant bacterial OTUs of wild and domesticated rice

**Additional file 10.** Differentially abundant fungal OTUs of wild and domesticated rice

**Additional file 11.** Degree and betweenness centrality of network nodes of wild rice, domesticated rice, and all rice

**Additional file 12.** Core OTUs based on prevalence

**Additional file 13.** Pedigree of rice cultivars developed in South Korea. Information on each cultivar is gathered from Nongsaro (http://www.nongsaro.go.kr) managed by the Rural Development Administration, Korea. Based on parental relationships, the pedigree was constructed using Helium (The James Hutton Institute, version 1.17.08.14). Cultivars used in this study were indicated as red circles for Japonica and orange circles for Tongil types. Each color of the lines indicates each breeding line (green, Dongjin breeding line; brown, IR 24 breeding line; purple, Koshihikari breeding line; orange, Nipponbare breeding line; blue, Nongrim 6 breeding line; dark red, Palgoeng breeding line; black, TN1 breeding line).

**Additional file 14.** Prediction on the functional pathways of bacterial communities in wild and domesticated rice

**Additional file 15.** Prediction on the functional redundancy of bacterial communities in wild and domesticated rice

**Additional file 16.** Relative abundance and ecological guilds of fungal OTUs associated with 43 rice accessions

## Data Availability

All raw sequences derived from this experiment were submitted into the Short Read Archive of NCBI and can be found under the BioProject accession number PRJNA532281. Metadata files, R data files, and R notebooks for full analyses are available from https://github.com/KiseokLee/RiceSeedMicrobiome.

## Abbreviations

CAP: Constrained analysis of principal coordinates
CSS: Cumulative sum scaling
ITS: Internal transcribed spacer
OTU: Operational taxonomic unit
PCo: Principal coordinate
PCoA: Principal coordinate analysis
PERMANOVA: Permutational multivariate analysis of variance
PNA: Peptide nucleic acid
RF: Random forest
rRNA: Ribosomal RNA
SAM: Shoot apical meristem
spp.: Species
