# Supplementary material for: Domestication of Oryza species eco-evolutionarily shapes bacterial and fungal communities in rice seed
Source: Microbiome. 2020 Feb 14;8:20. doi: 10.1186/s40168-020-00805-0 (PMC7023700; doi:10.1186/s40168-020-00805-0)
Supplement: Supplementary file 2 — Additional file 1: Figure S1. Abundance profiles depending on different normalization and transformation methods. The frequency distribution of OTUs was plotted to compare the effects of normalization methods. CSS normalization with and without log2(1+x) transformation, rarefying by 1,000 reads, relative abundance, and Hellinger transformation (square root of relative abundance) methods were tested. CSS normalization with log2(1+x) transformation and Hellinger transformation had distributions close to Gaussian distribution. CSS, cumulative sum scaling. Figure S2. The use of Peptide Nucleic Acid (PNA) clamps during the PCR step reduced mitochondrial and plastid DNA contamination from the rice plant. (a) Usable reads in raw sequence reads of all sample replicates. ‘Usable reads’ (light blue) are reads that excluded Chloroplast (light green) and Mitochondrial (orange) reads from the raw reads. With the use of PNA clamps more than about 10,000 reads were usable per sample replicate, whereas without the use of PNA clamps much less reads were usable. We compared the resolution of taxonomic identification in relative abundance (RA) bar plots at the (b) family level and (c) genus level of bacterial taxa of 43 rice accessions’ seeds. High abundance taxonomic groups did not differ between with and without PNA clamps, but low abundance taxonomic groups were more identified in the PNA clamp-used samples at both the family and genus level. Taxonomic groups with less than 5 ‰ (per-mille) of each samples were labeled as ‘Low abundance’. Each technical replicate comprised a pool of three sets of three grains. Further statistical information on average reads, OTUs and Shannon diversity index, is detailed in Additional file 5. Abbreviations for rice accessions are available in Table S1. Figure S3. Relative abundance (RA) in the (a) phylum, (b) class and (c) order level of bacterial taxa in the seeds of 43 rice accessions. Low abundance taxonomic groups with less than 5 ‰ (per-mille) [file 40168_2020_805_MOESM1_ESM.docx]

**Additional file 1:**

**Domestication of *Oryza* species eco-evolutionarily shapes bacterial and fungal communities in rice seed**

**Hyun Kim^1†^, Kiseok Keith Lee^1†^, Jongbum Jeon^2^, William Anthony Harris ^1^, Yong-Hwan Lee^1,2,3,4,5*^**

^1^Department of Agricultural Biotechnology, ^2^Interdisciplinary Program in Agricultural Genomics, ^3^Center for Fungal Genetic Resources, ^4^Plant Immunity Research Center, and ^5^Research Institute of Agriculture and Life Sciences, Seoul National University, Seoul 08826, Korea

*Corresponding author: yonglee@snu.ac.kr

^†^These authors contributed equally to this work.

**Additional file 1 contains:**

Supporting information

Figure S1-S26

Table S1-S4

References

**Supporting information**

**Functional profiling reveals domestication effects on bacterial communities**

To unveil the effect of domestication on the functions of seed microbial communities, we performed a functional prediction on the bacterial community using Tax4Fun2 (Additional file 14). Although the deduction of microbial functions from the partial sequences of 16S rRNA genes has to be construed carefully, the bacterial community associated with wild rice was enriched in nitrogen and lipid metabolisms. On the other hand, the bacterial community in domesticated rice was enriched in the metabolisms of carbohydrates and vitamins. Domesticated rice seeds are reported to have a higher carbohydrate content and a lesser protein content in grains compared to wild rice seeds [1]. The enriched metabolisms in bacterial community of domesticated rice could be correlated with the nutritional changes in rice seed during domestication (Additional file 14). Together, these results indicate that the introduction of new agronomic traits can affect the predicted functions of bacterial community in domesticated rice. We also investigated the functional redundancy to estimate potential functional stability. The functional redundancy of 2,567 out of 3,236 functions was significantly higher in domesticated rice than wild one (Additional file 15). These results suggest that the composition of bacterial community of domesticated rice are more stable than that of wild rice.

In order to understand the responses of fungal communities to the domestication in the ecological aspect, distribution of fungal guilds was investigated using FunGuild. Individual rice accessions possessed distinct guild compositions (Additional file 1: Figure S26a; Additional file 16). Putative plant pathogens and undefined saprotrophs were more abundant in wild rice seeds, whereas the guild ‘Animal pathogen-Plant pathogen-Undefined saprotroph’ was more distributed in domesticated rice seeds (Additional file 1: Figure S26b-f). However, there is the limitation on understanding the difference in functionality of fungal communities because the information on functional genes is lacked.

**Figure S1**


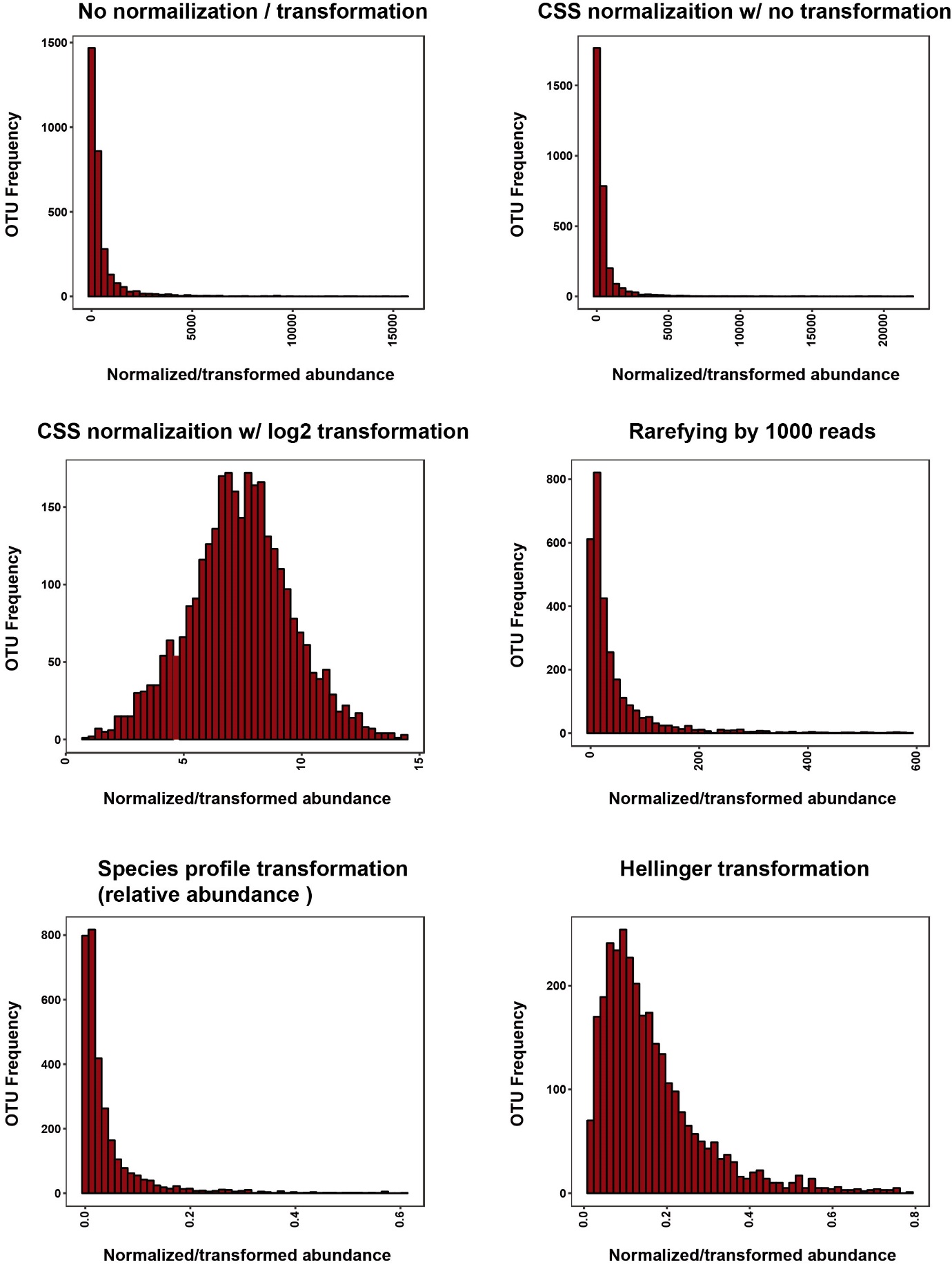


**Figure S1. Abundance profiles depending on different normalization and transformation methods.** The frequency distribution of OTUs was plotted to compare the effects of normalization methods. CSS normalization with and without log2(1+x) transformation, rarefying by 1,000 reads, relative abundance, and Hellinger transformation (square root of relative abundance) methods were tested. CSS normalization with log2(1+x) transformation and Hellinger transformation had distributions close to Gaussian distribution. CSS, cumulative sum scaling.

**Figure S2**


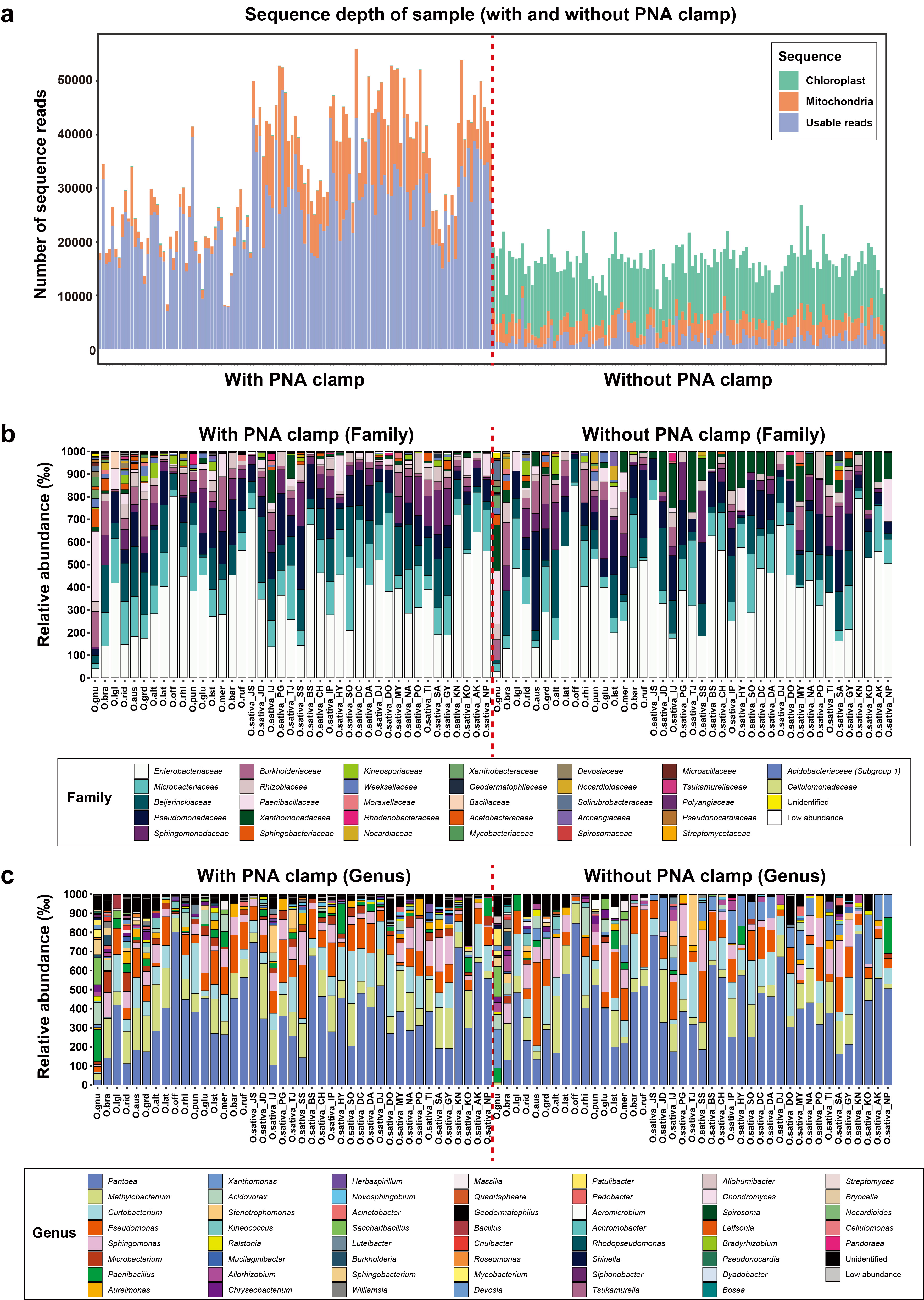


**Figure S2. The use of Peptide Nucleic Acid (PNA) clamps during the PCR step reduced mitochondrial and plastid DNA contamination from the rice plant.** (a) Usable reads in raw sequence reads of all sample replicates. ‘Usable reads’ (light blue) are reads that excluded Chloroplast (light green) and Mitochondrial (orange) reads from the raw reads. With the use of PNA clamps more than about 10,000 reads were usable per sample replicate, whereas without the use of PNA clamps much less reads were usable. We compared the resolution of taxonomic identification in relative abundance (RA) bar plots at the (b) family level and (c) genus level of bacterial taxa of 43 rice accessions’ seeds. High abundance taxonomic groups did not differ between with and without PNA clamps, but low abundance taxonomic groups were more identified in the PNA clamp-used samples at both the family and genus level. Taxonomic groups with less than 5 ‰ (per-mille) of each samples were labeled as ‘Low abundance’. Each technical replicate comprised a pool of three sets of three grains. Further statistical information on average reads, OTUs and Shannon diversity index, is detailed in Additional file 5. Abbreviations for rice accessions are available in Table S1.

**Figure S3**


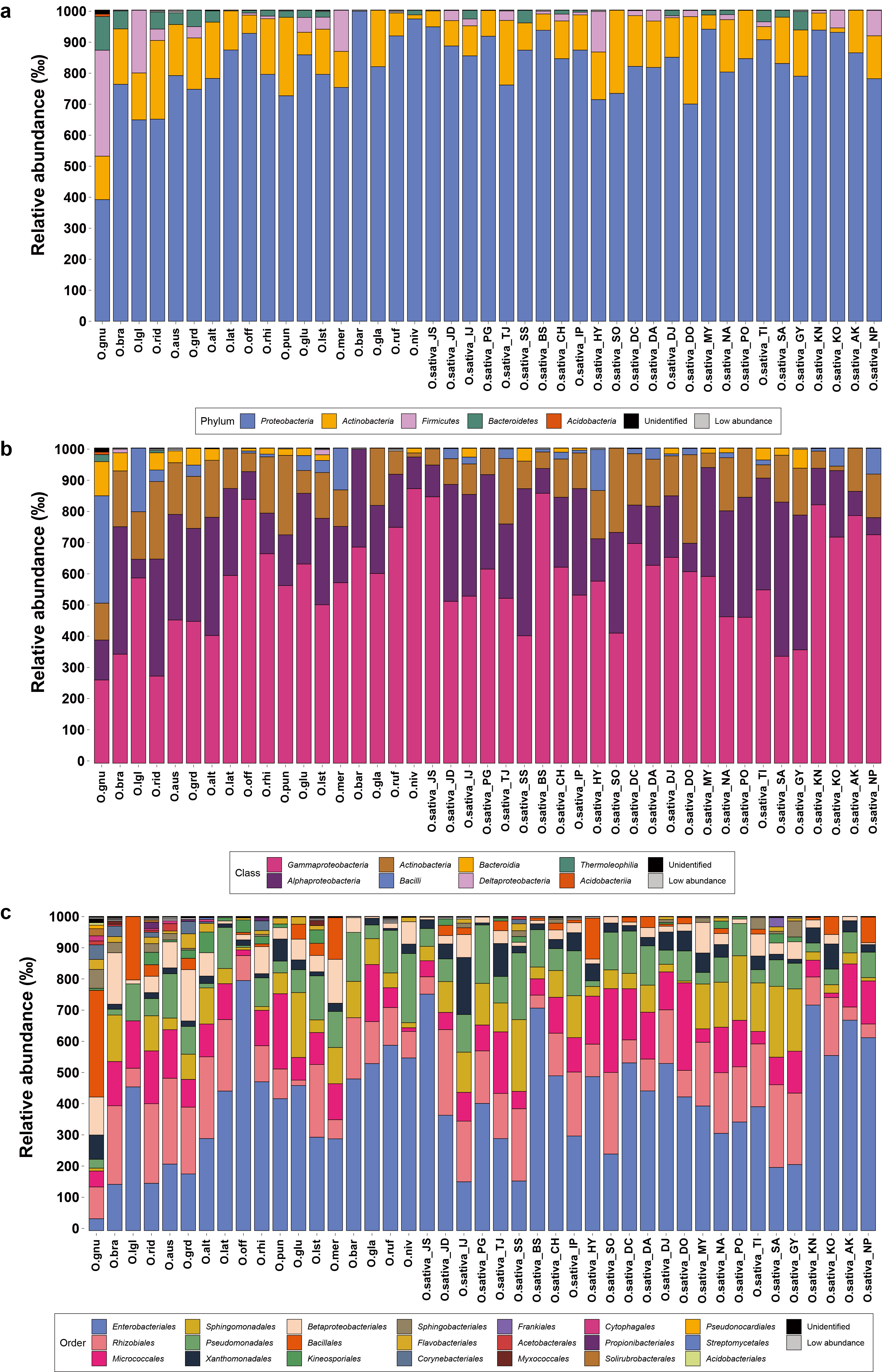


**Figure S3. Relative abundance (RA) in the (a) phylum, (b) class and (c) order level of bacterial taxa in the seeds of 43 rice accessions.** Low abundance taxonomic groups with less than 5 ‰ (per-mille) of each samples are highlighted in gray. Unidentified taxonomic groups are indicated in black. Each technical replicate comprised a pool of three sets of three grains. Abbreviations for rice accessions are available in Table S1.

**Figure S4**


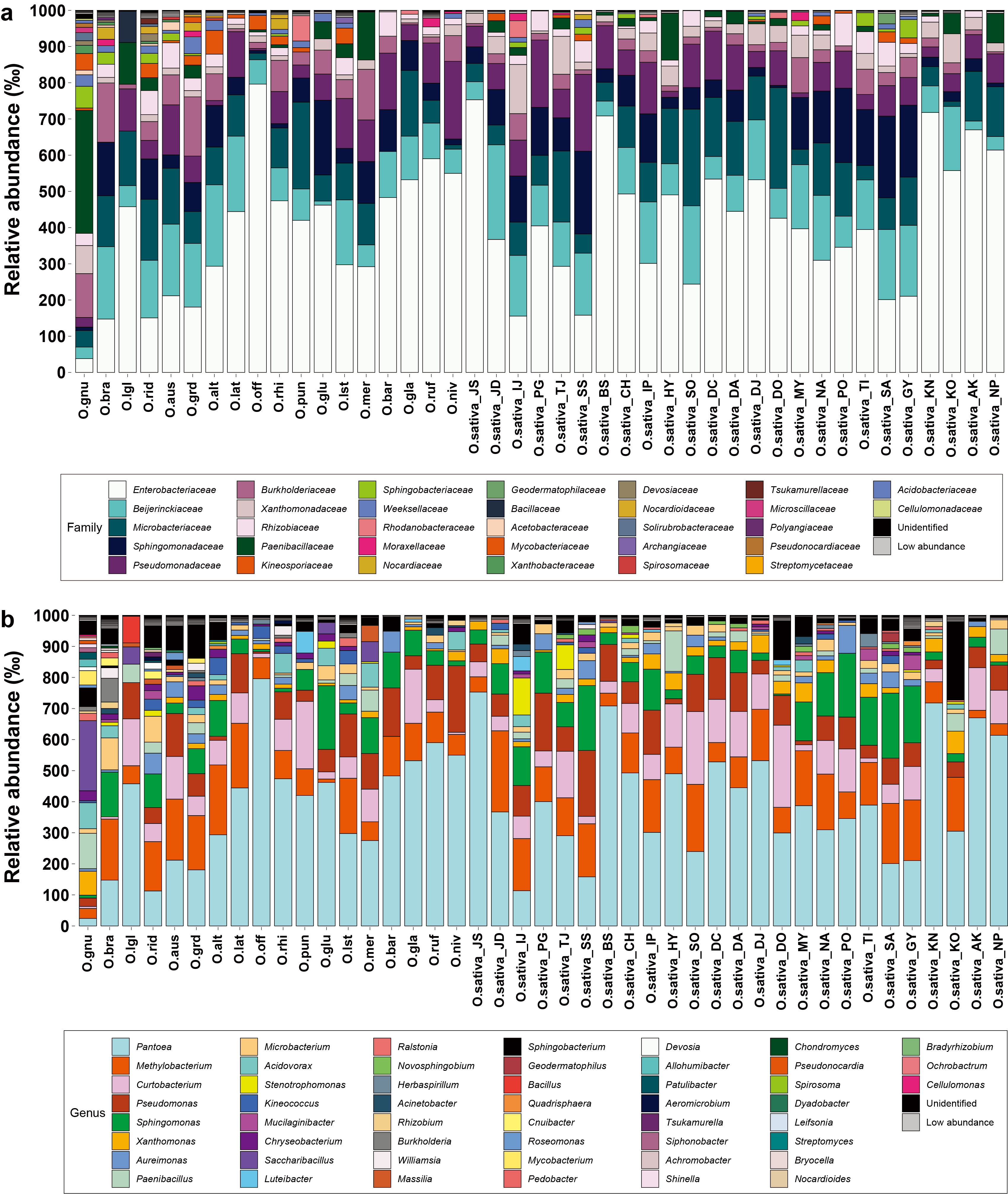


**Figure S4. Relative abundance (RA) in the (a) family and (b) genus level of bacterial taxa in the seeds of 43 rice accessions.** Low abundance taxonomic groups with less than 5 ‰ (per-mille) of each samples are highlighted in gray. Unidentified taxonomic groups are indicated in black. Each technical replicate comprised a pool of three sets of three grains. Abbreviations for rice accessions are available in Table S1.

**Figure S5**


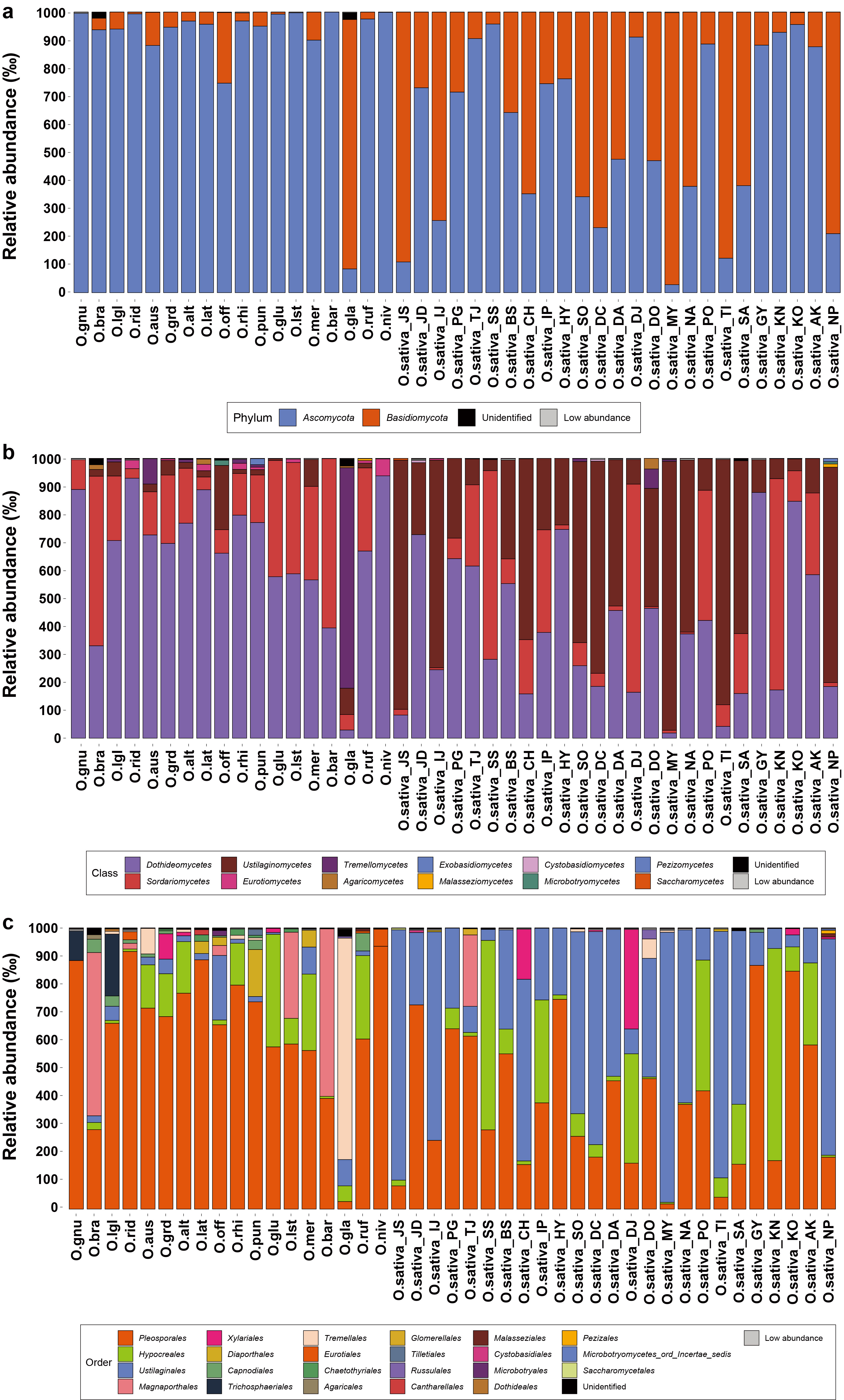


**Figure S5. Relative abundance (RA) in the (a) phylum, (b) class, and (c) order level of fungal taxa in the seeds of 43 rice accessions.** Low abundance taxonomic groups with less than 5 ‰ (per-mille) of each samples are highlighted in gray. Unidentified taxonomic groups are indicated in black. Each technical replicate comprised a pool of three sets of three grains. Abbreviations for rice accessions are available in Table S1.


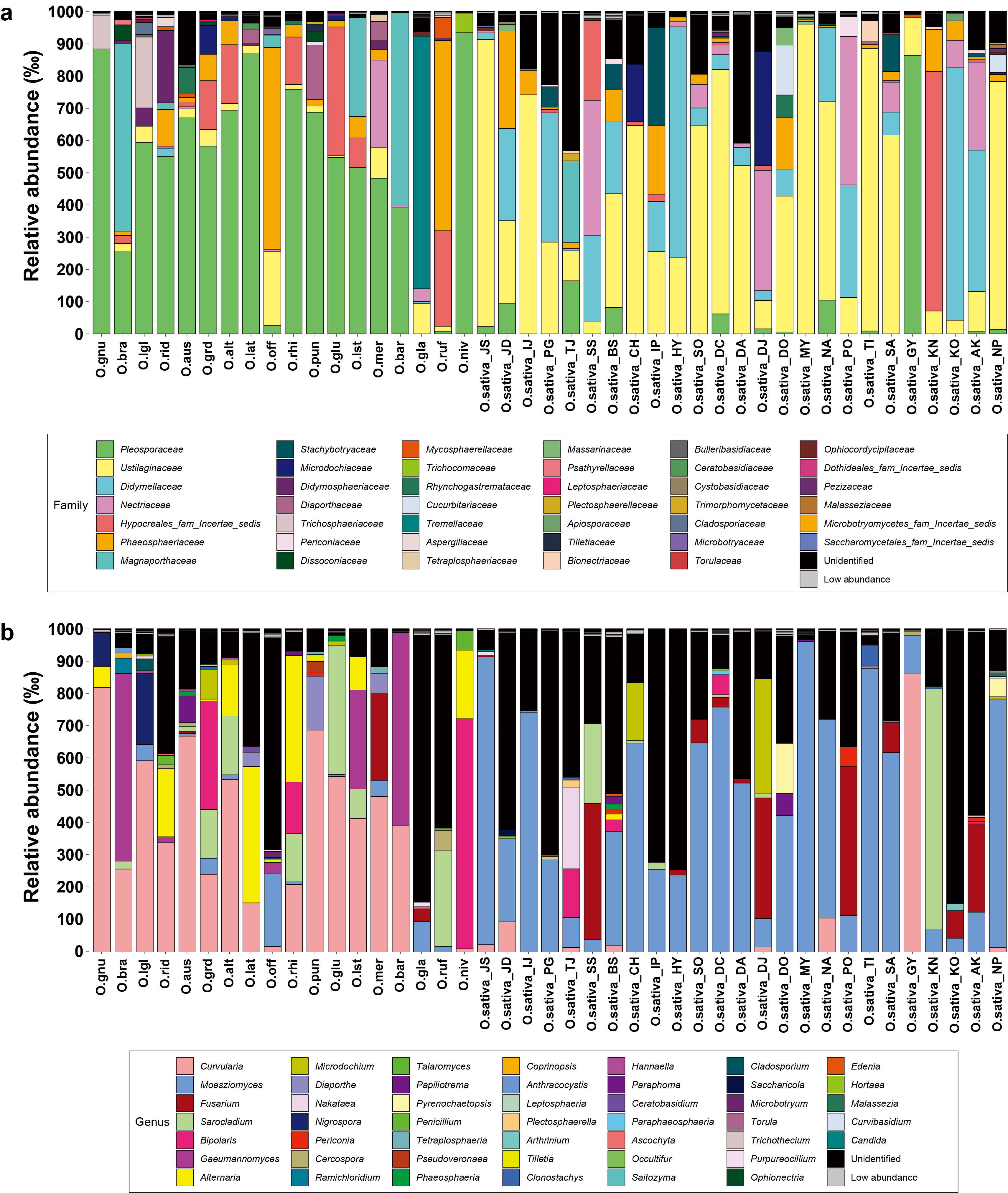


**Figure S6. Relative abundance (RA) in the (a) family and (b) genus level of fungal taxa in the seeds of 43 rice accessions.** Low abundance taxonomic groups with less than 5 ‰ (per-mille) of each samples are highlighted in gray. Unidentified taxonomic groups are indicated in black. Each technical replicate comprised a pool of three sets of three grains. Abbreviations for rice accessions are available in Table S1.


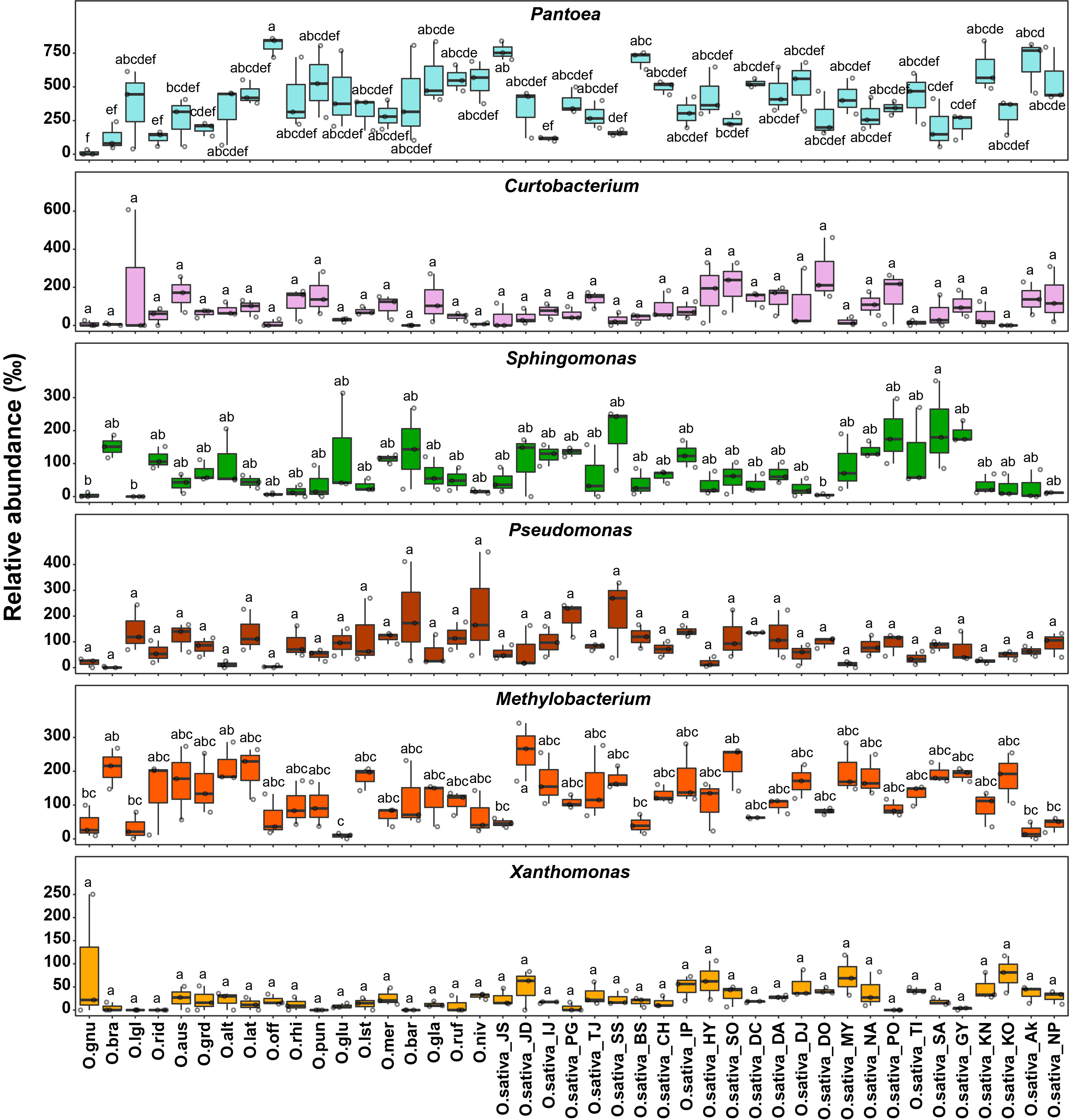


**Figure S7. Relative abundance of major genera of bacterial biota.** Six major genera in bacterial biotas are selected based on the total sequence reads. Statistically significant differences between group means of relative abundance were determined by one-way ANOVA (P<0.05). Three replicates per an accession were used. Different letters indicate statistically significant differences. Abbreviations for rice accessions are available in Table S1.


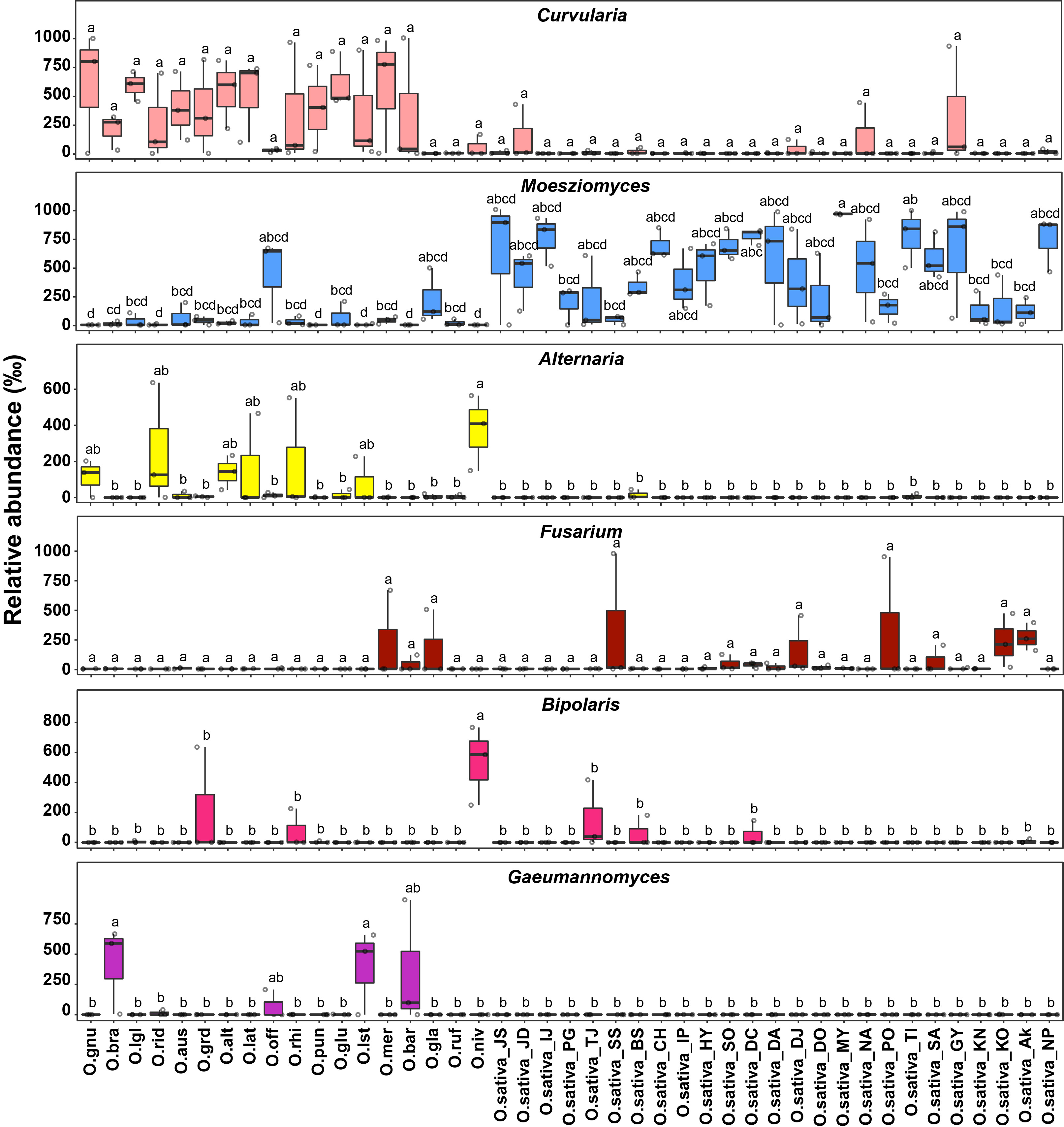


**Figure S8. Relative abundance of major genera of fungal biota.** Six major genera in fungal biota are selected based on the total sequence reads. Statistically significant differences between group means of relative abundance were determined by one-way ANOVA (P<0.05). Three replicates per accession were used. Different letters indicate statistically significant differences. Abbreviations for rice accessions are available in Table S1.


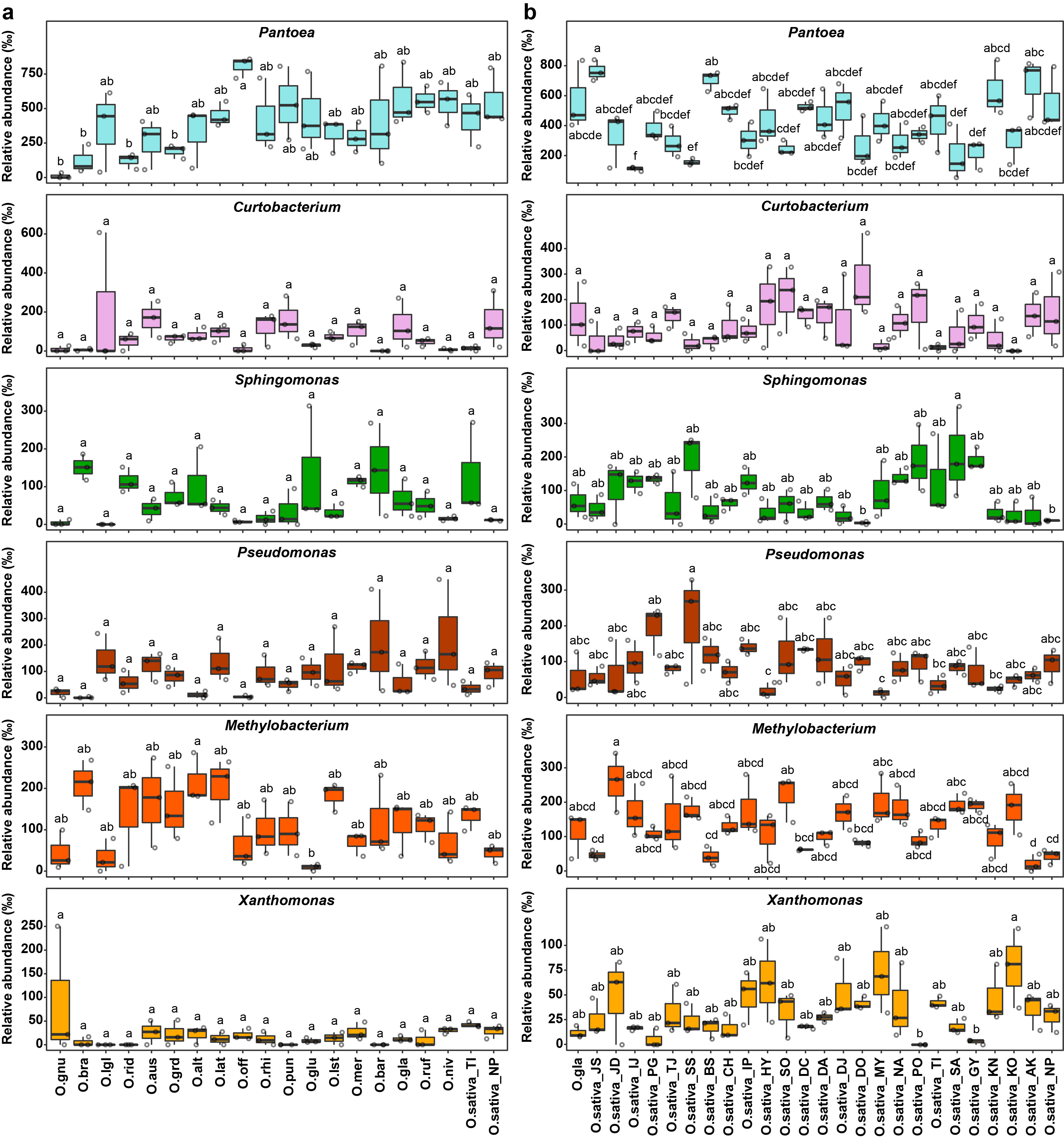


**Figure S9. Comparing relative abundance of major genera of bacteria between domesticated and wild rice accessions.** Six major genera of bacterial biota are selected based on the total sequence reads in wild and domesticated rice accessions, respectively. Statistically significant differences between group means of relative abundance were determined by one-way ANOVA (P<0.05). Three replicates per accession were used. Different letters indicate statistically significant differences. Abbreviations for rice accessions are available in Table S1.


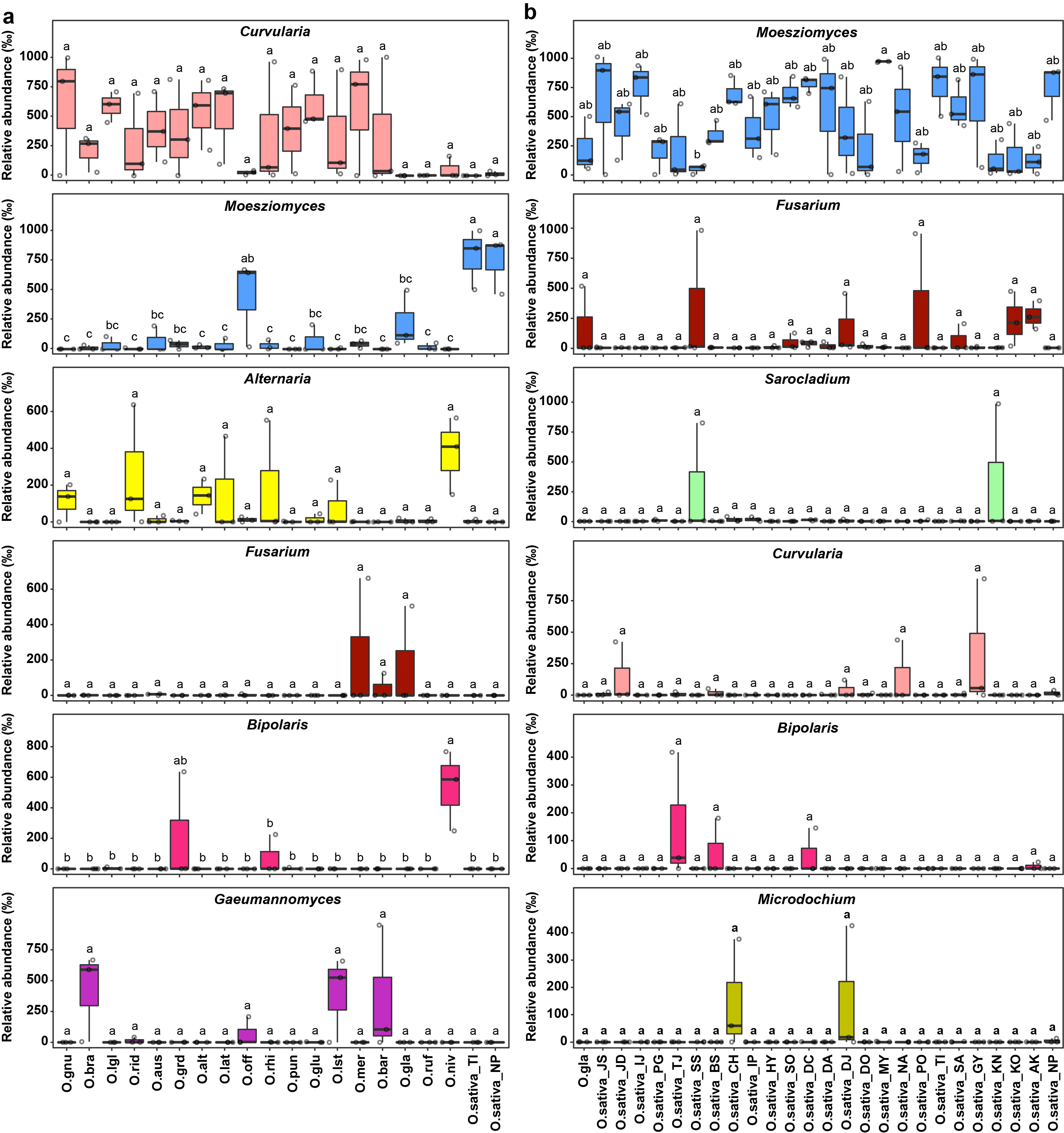


**Figure S10. Comparing relative abundance of major genera of fungi between domesticated and wild rice accessions.** Six major genera in fungal biota are selected based on the total sequence reads in wild and domesticated rice accessions, respectively. Statistically significant differences between group means of relative abundance were determined by one-way ANOVA (P<0.05). Three replicates per accession were used. Different letters indicate statistically significant differences. Abbreviations for rice accessions are available in Table S1.


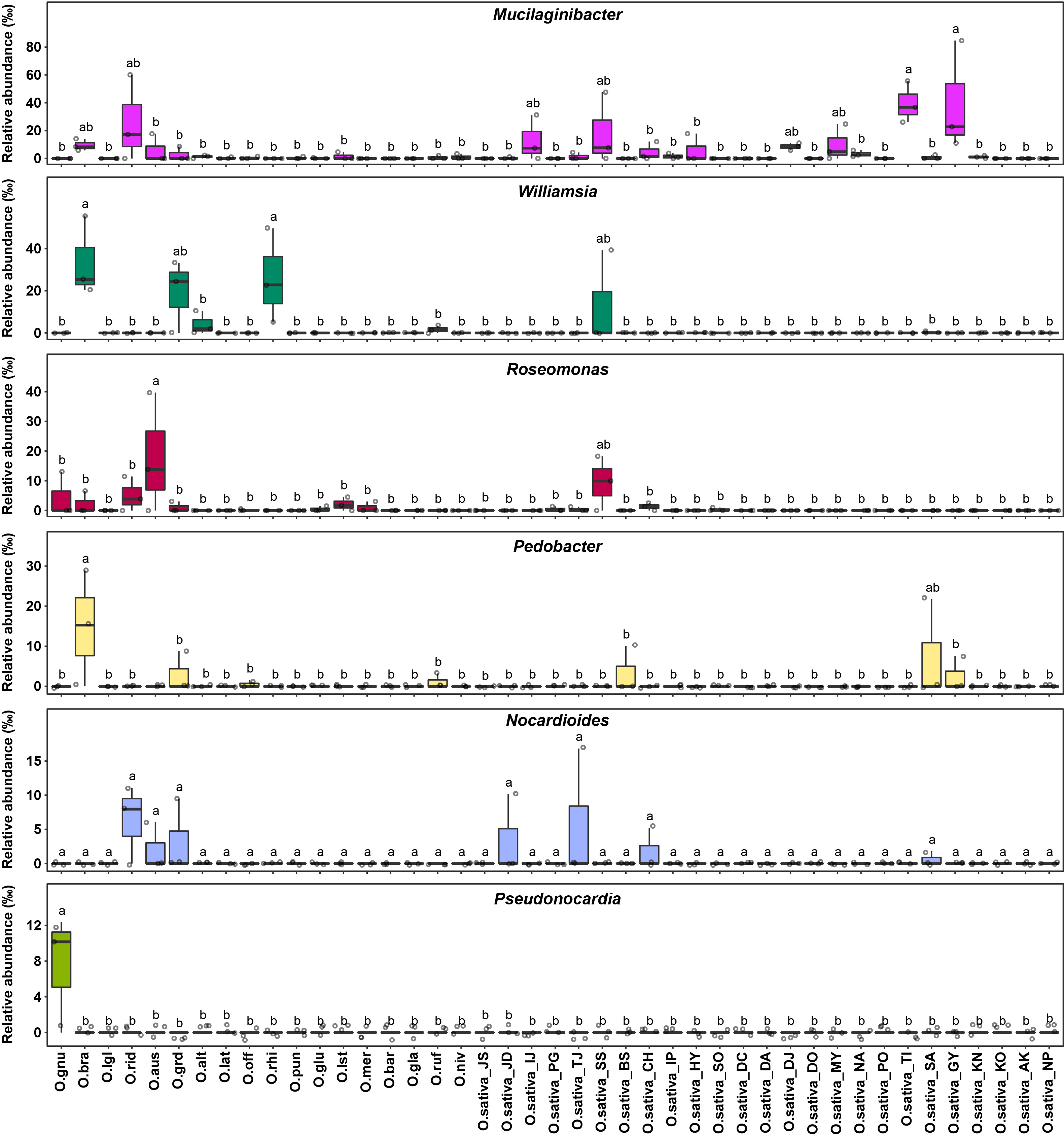


**Figure S11. Comparing relative abundance of minor genera of bacterial biota among rice accessions.** Six minor genera in bacterial biota are selected based on the total sequence reads. Statistically significant differences between group means of relative abundance were determined by one-way ANOVA (P<0.05). Three replicates per accession were used. Different letters indicate statistically significant differences. Abbreviations for rice accessions are available in Table S1.


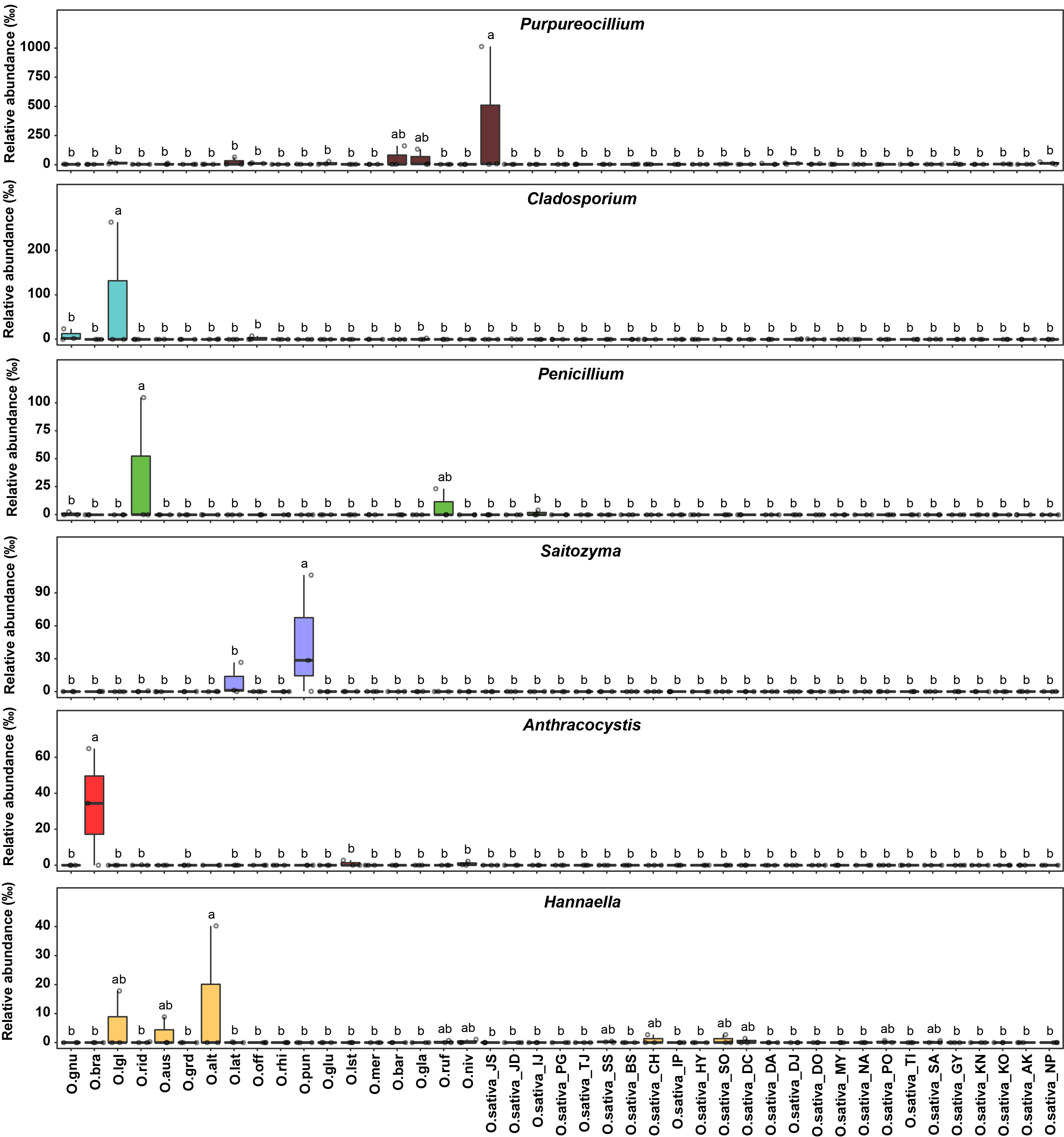


**Figure S12. Comparing relative abundance of minor genera of fungal biota among rice accessions.** Six minor genera in fungal biota are selected based on the total sequence reads. Statistically significant differences between group means of relative abundance were determined by one-way ANOVA (P<0.05). Three replicates per accession were used. Different letters indicate statistically significant differences. Abbreviations for rice accessions are available in Table S1.

**Figure S13**


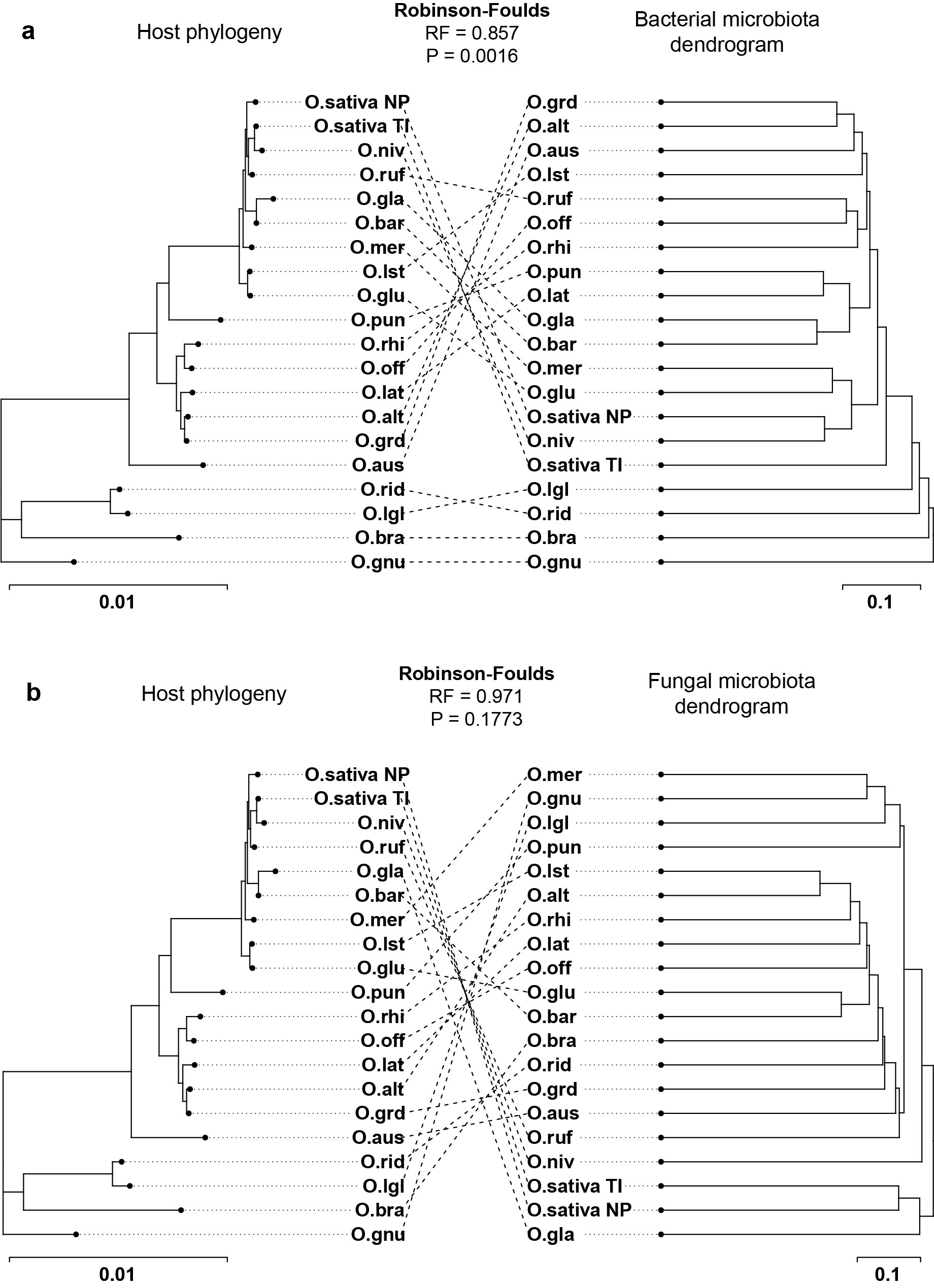


**Figure S13. Cophylogenetic tree of *Oryza* species and dendrogram of hierarchical clustering of Bray-Curtis distance of (a) bacterial and (b) fungal microbiotas.** The phylogenetic tree of 17 *Oryza* spp. and 2 *O. sativa* cultivars (Tongil and Nipponbare). The tree was constructed based on the chloroplast genome sequence. RAxML program was used to draw Maximum Likelihood (ML) tree with 1,000 bootstraps. Dendrograms of bacterial and fungal biotas were constructed based on hierarchical clustering of Bray-Curtis distance using hclust() in R. Cophylogenetic trees were generated using the cophylo() in ‘phytools’ package. Robinson-Foulds score was calculated based on 100,000 randomized trees to quantify the topological congruency of host phylogeny and dendrograms of bacterial and fungal biotas. Bacterial biota showed significant eco-evolutionary relationships with rice phylogeny (RF = 0.857, P = 0.0016).


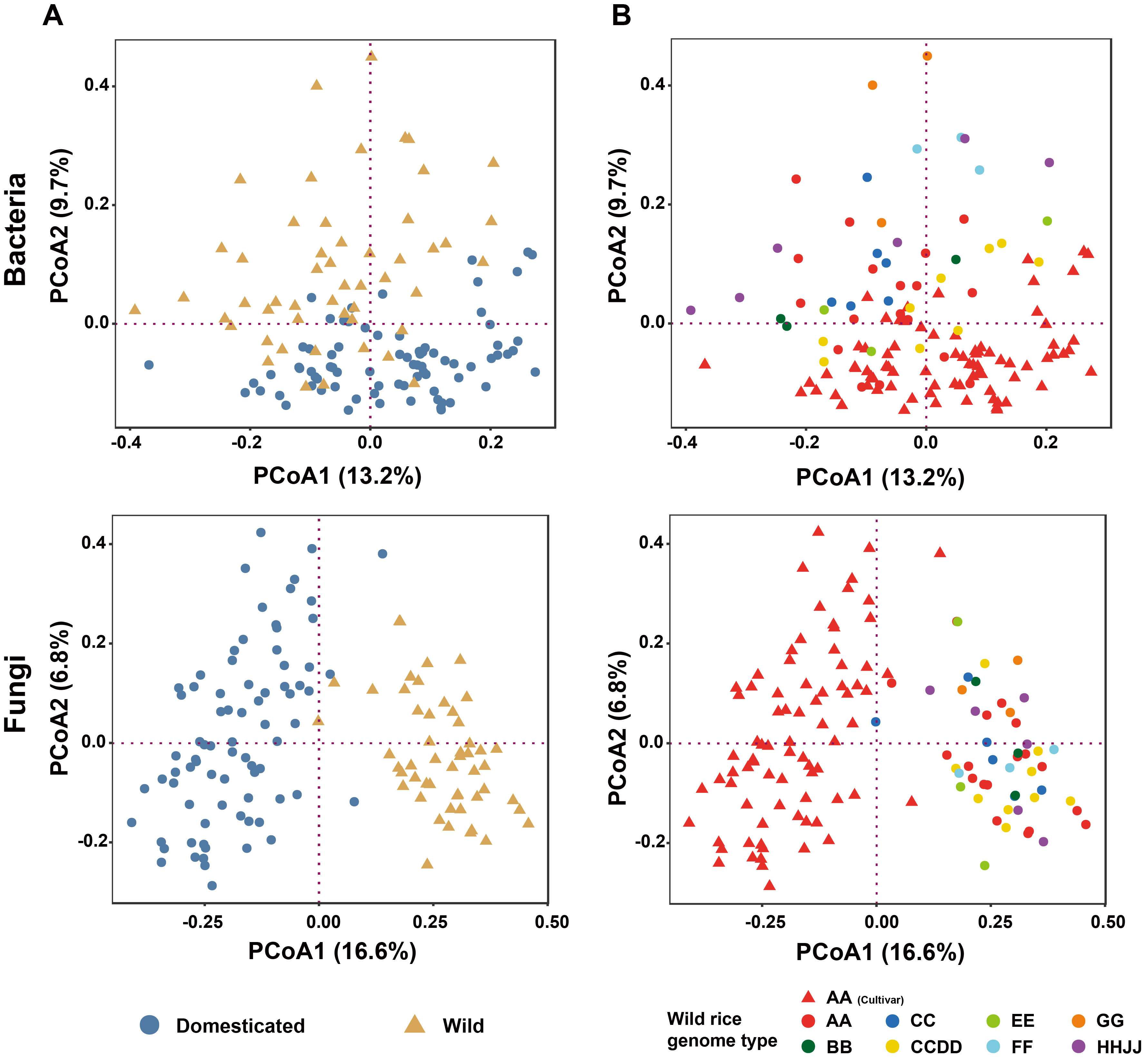


**Figure S14. Unconstrained principal coordinate ordination (PCoA) of the seed microbial communities.** PCoA analysis of bacterial and fungal diversity in the seed endosphere was done with pairwise Bray-Curtis distances of all 43 rice accessions used in this study. CSS/log transformed reads were used to calculate Bray–Curtis distances. Each point represents each sample replicates and was colored by (a) wild and domesticated rice categorization and (b) rice genome type (AA, BB, CC, CCDD, EE, FF, GG, and HHJJ).

**Figure S15**


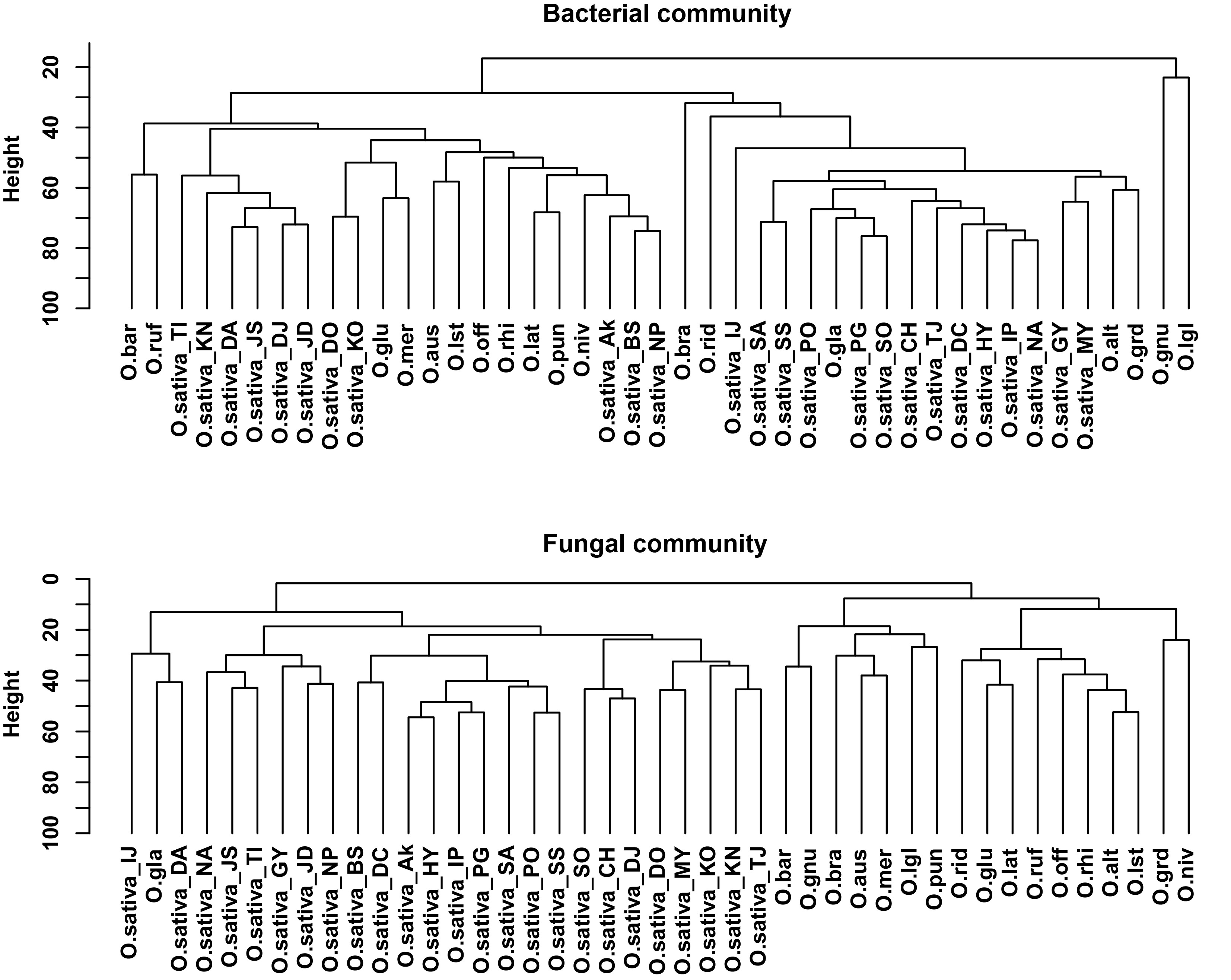


**Figure S15. Dendrogram of bacterial and fungal communities of 43 rice accessions based on Bray-Curtis distance.** The dendrograms of bacterial and fungal communities were constructed using Bray-Curtis distance. Upper and lower panels indicate bacterial and fungal communities, respectively. The abbreviations of each accession are available in Table S1.


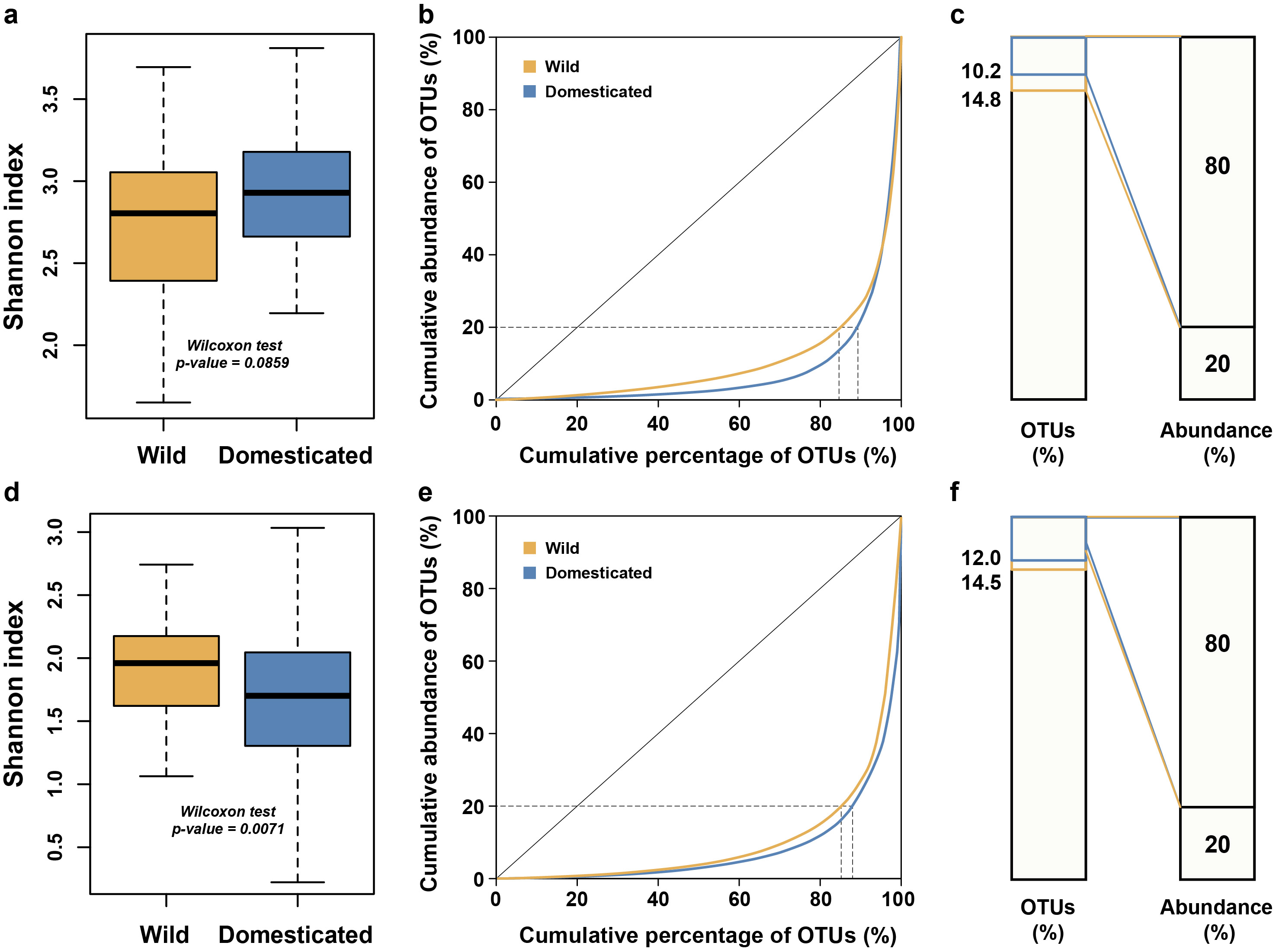


**Figure S16. Domestication effects on alpha diversity and community structure measured by cumulative abundance and percentage of OTUs.** (a and d) Alpha diversity Shannon index comparing wild and domesticated rice seed (a) bacterial and (d) fungal communities. Wild rice includes 17 rice species and domesticated rice includes 25 *O*. *sativa* cultivars and *O*. *glaberrima*. (b and e) Cumulative abundance of total abundance (Y-axis) plotted against cumulative percentage of OTUs from the lowest to highest abundance (X-axis) in (b) bacterial and (e) fungal biotas. This graph (Lorenz-curve) was plotted to show how much the community abundance was dominated by few OTUs in wild and domesticated rice seed microbiome. The larger the area between the curve and the diagonal line, the higher the inequality. (c and f) Diagram show the percentage of total OTUs accounting for 80% of the total abundance (normalized sequence reads) in (c) bacterial and (f) fungal biotas. This diagram demonstrates the Pareto rule (80-20 principle) in the seed microbiome and compares wild rice to their domesticated counterpart.

**Figure S17**


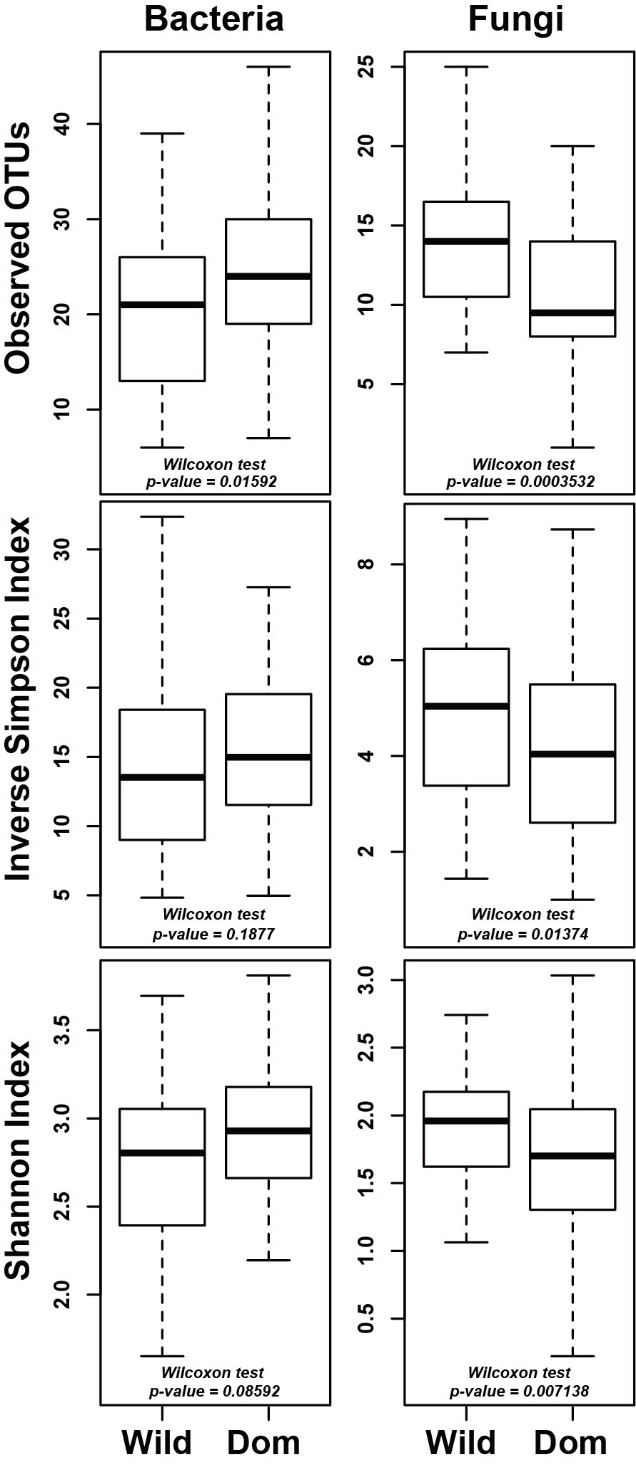


**Figure S17. Alpha diversity comparison of wild and domesticated rice seed microbiota.** Observed OTUs, inverse Simpson index, and Shannon index of 17 wild rice accessions and 26 domesticated rice accessions were calculated after Hellinger transformation of OTU tables. In bacteria, the number of observed OTUs was significantly higher in domesticated rice seeds than the wild counterpart (Wilcoxon rank-sum test, P < 0.05) but the differences in inverse Simpson and Shannon indices were not significant. In fungi, the number of observed OTUs, inverse Simpson and Shannon indices were significantly higher in wild rice seeds than the domesticated counterpart (Wilcoxon rank-sum test, P < 0.05). Wild, wild rice; Dom, domesticated rice.


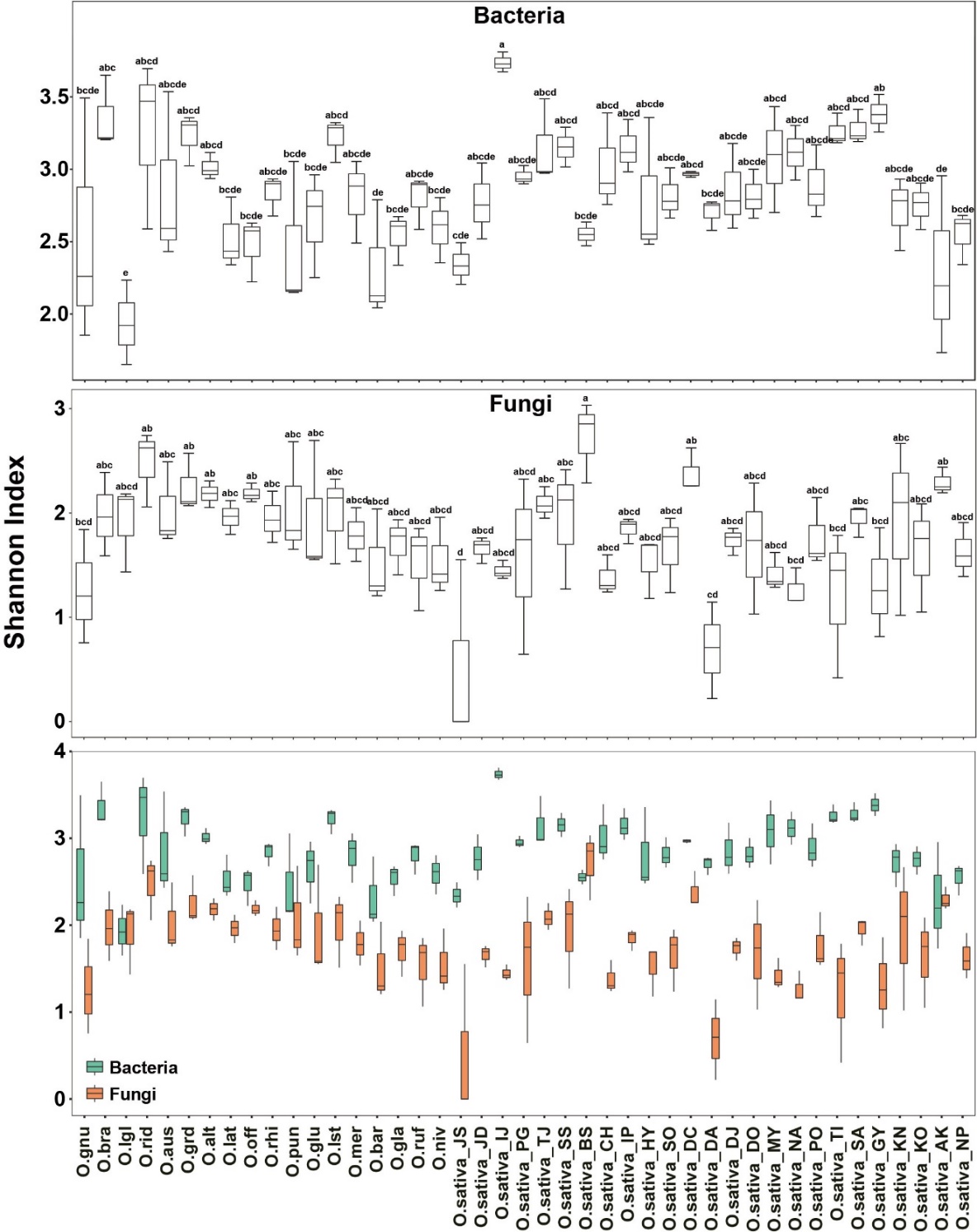


**Figure S18. Alpha diversity comparison of all 43 rice accession seed bacterial and fungal biotas.** The comparison of Shannon index between bacteria and fungi of each rice accessions shows that bacterial Shannon index (green) tends to be higher than fungal Shannon index (orange) in each rice seed microbial communities. Shannon index were calculated after Hellinger transformation of bacterial and fungal OTU tables. Abbreviations for rice accessions are available in Table S1.

**Figure S19**


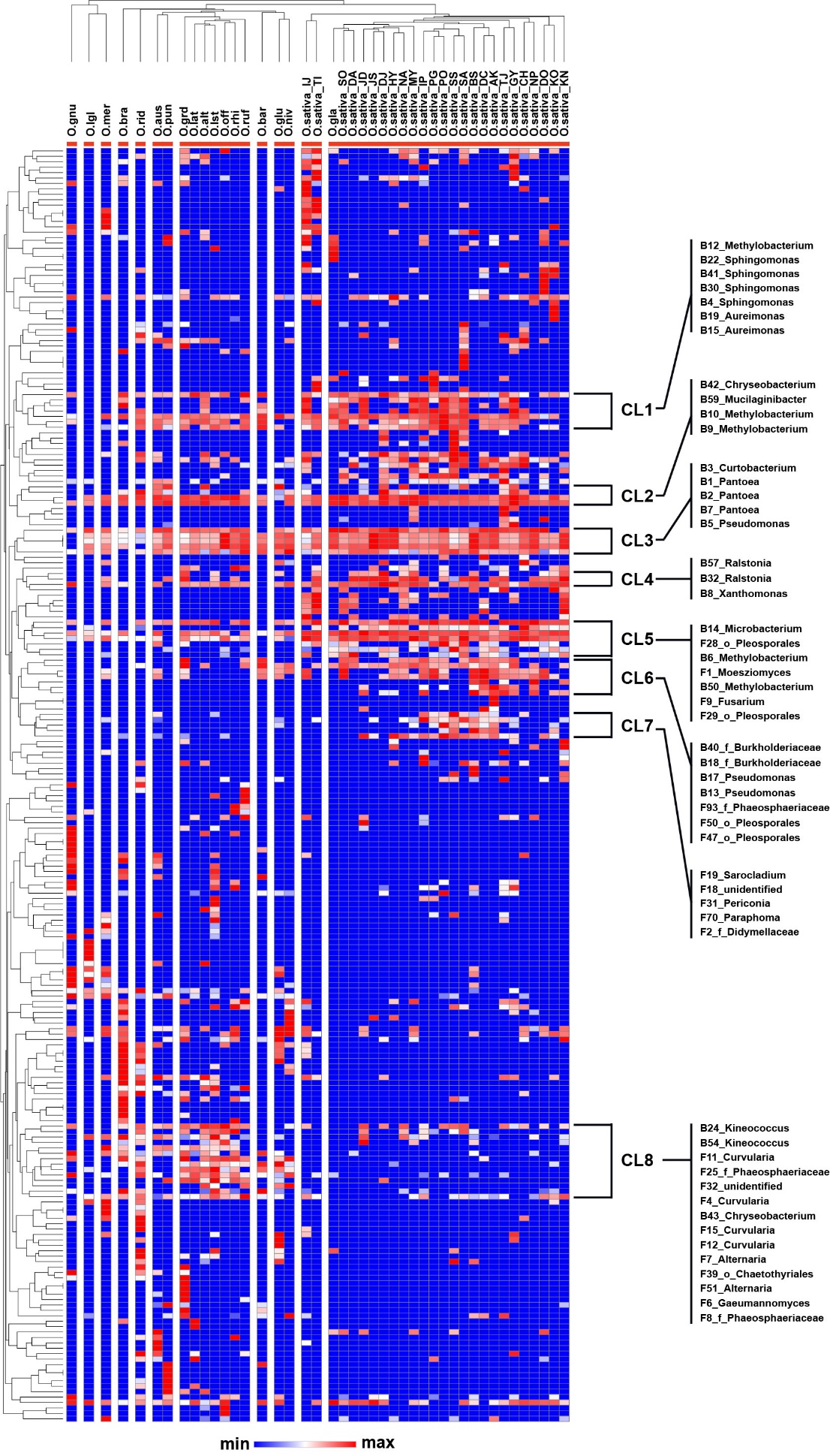


**Figure S19. Heatmap shows a distinct pattern of wild and domesticated rice seed colonizers.** Bacterial and fungal OTUs with more than 200 reads in the total sequence (127 bacterial OTUs, 108 fungal OTUs) were merged after CSS/log normalization of read counts. Samples and OTUs were hierarchically grouped (group-average linkage) based on the pairwise Spearman correlations both row-wise and column-wise. Hierarchical clustering and visualization was done on the Morpheus web platform (https://software.broadinstitute.org/morpheus).

**Figure S20**


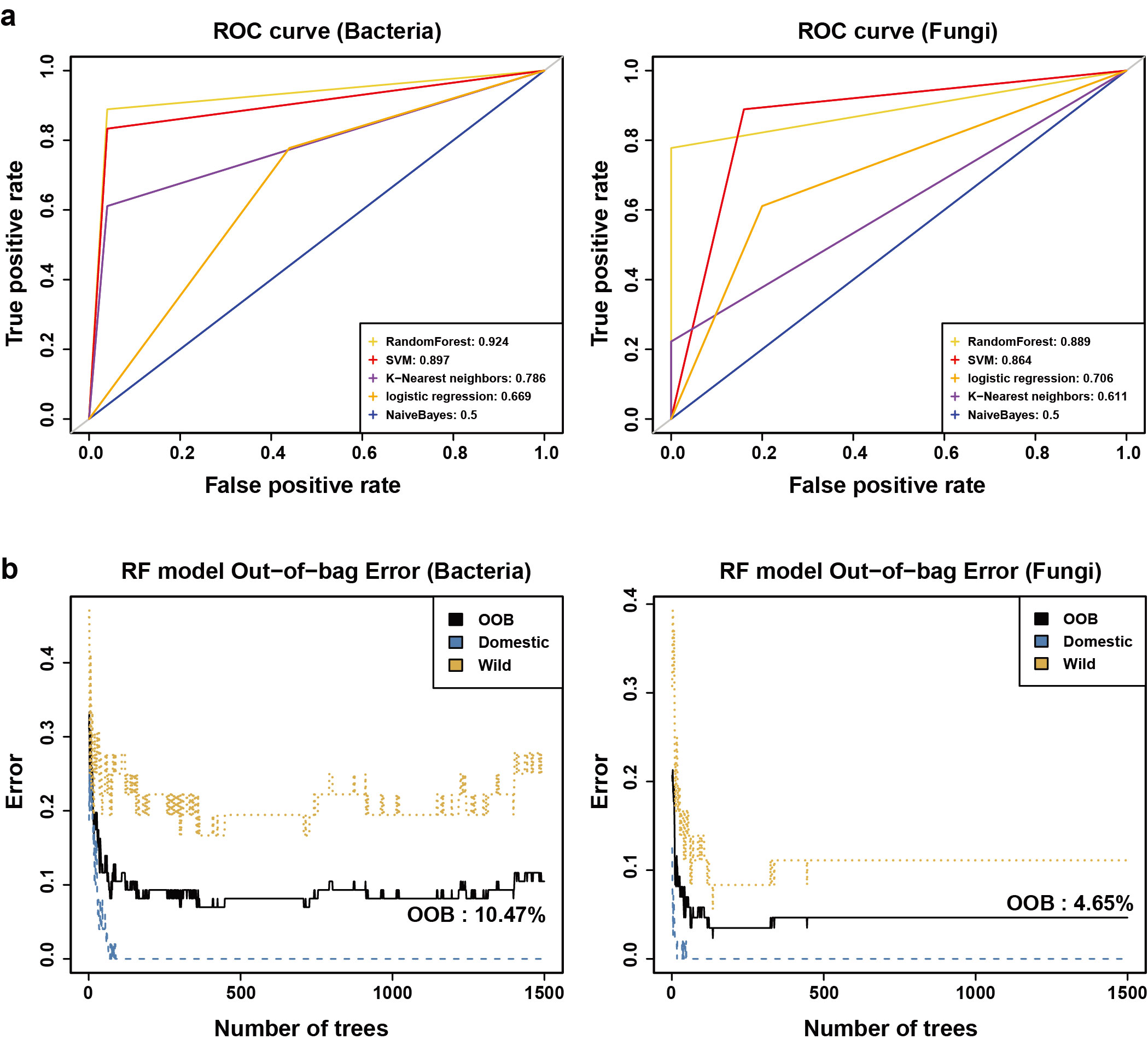


**Figure S20. ROC curve and Out-of-bag error rate of full Random Forest (RF) model.** Wild and domesticated rice category was set as dependent variables to be predicted with OTUs in 129 samples in 43 rice accessions (3 replicates). OTU tables were CSS/log normalized. Two-thirds of the samples of total samples were randomly sampled for the training set. (a) ROC curves were plotted with the remaining test set in order to test which machine learning algorithm had the best performance among 5 classifications: Support vector machine (SVM) Naïve Bayes, k-nearest neighbors, logistic regression. The number of trees for RF model was set to 1,500. Area under ROC curve (AUC) was calculated to measure 5 classifier’s performance and Random Forest model performed best (bacteria, AUC=0.924; fungi, AUC=0.889). (b) Out-of-bag error rate of RF models according to the number of trees was used. We used 1,500 trees which were in the range having the lowest Out-of-bag error rate.


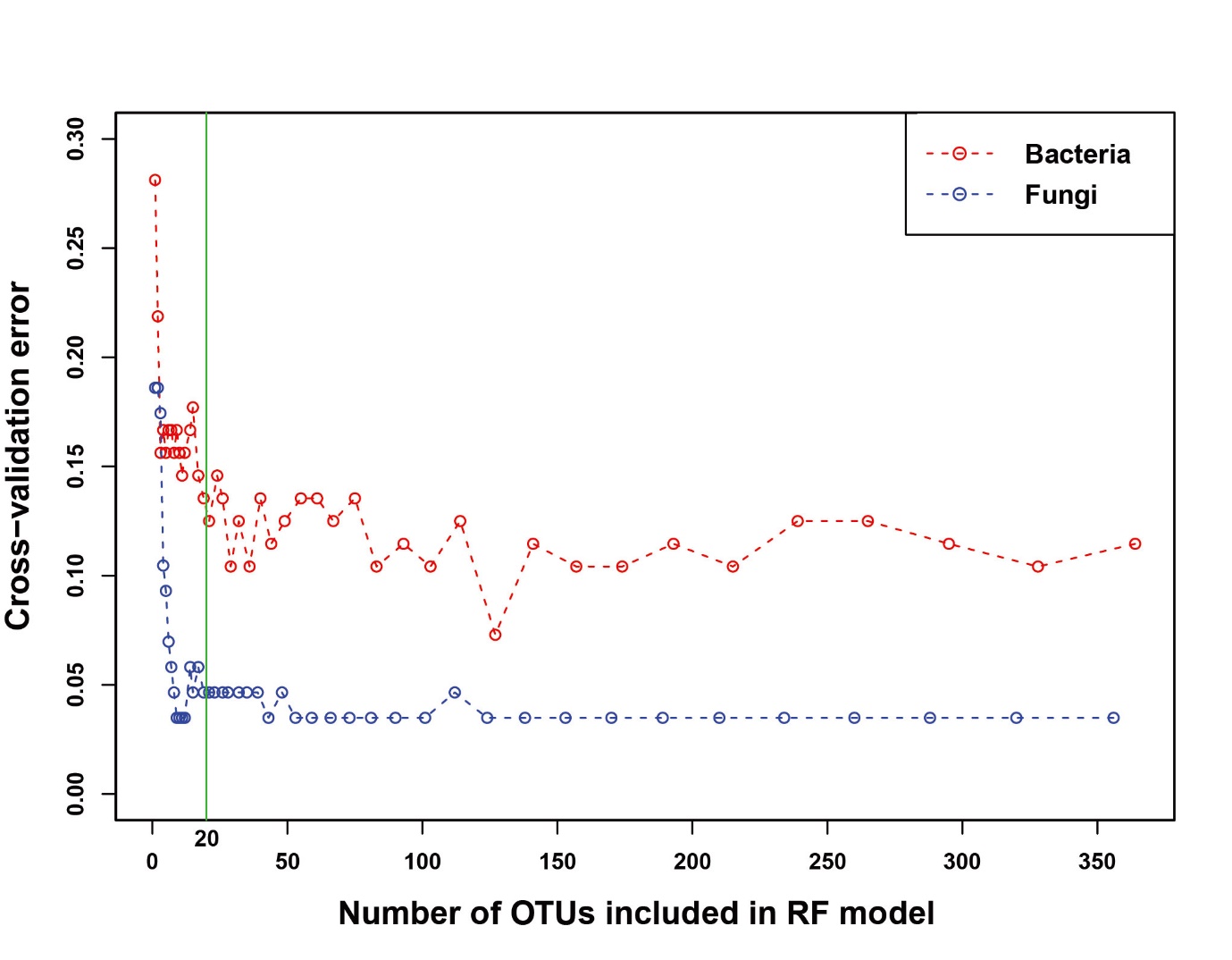


**Figure S21. Cross-validation error of Random Forest (RF) model with differing numbers of OTUs to predict wild and domesticated rice categories.** To evaluate model performance as a function of inclusion of the top wild/domesticated rice-discriminating OTUs, 10-fold cross validation was performed while gradually excluding less important OTUs. The prediction error rate was kept low from 364 bacterial and 356 fungal OTUs to approximately 20 OTUs. A rapid increase in the prediction error rate occurred when less than 20 of the most important OTUs were included.


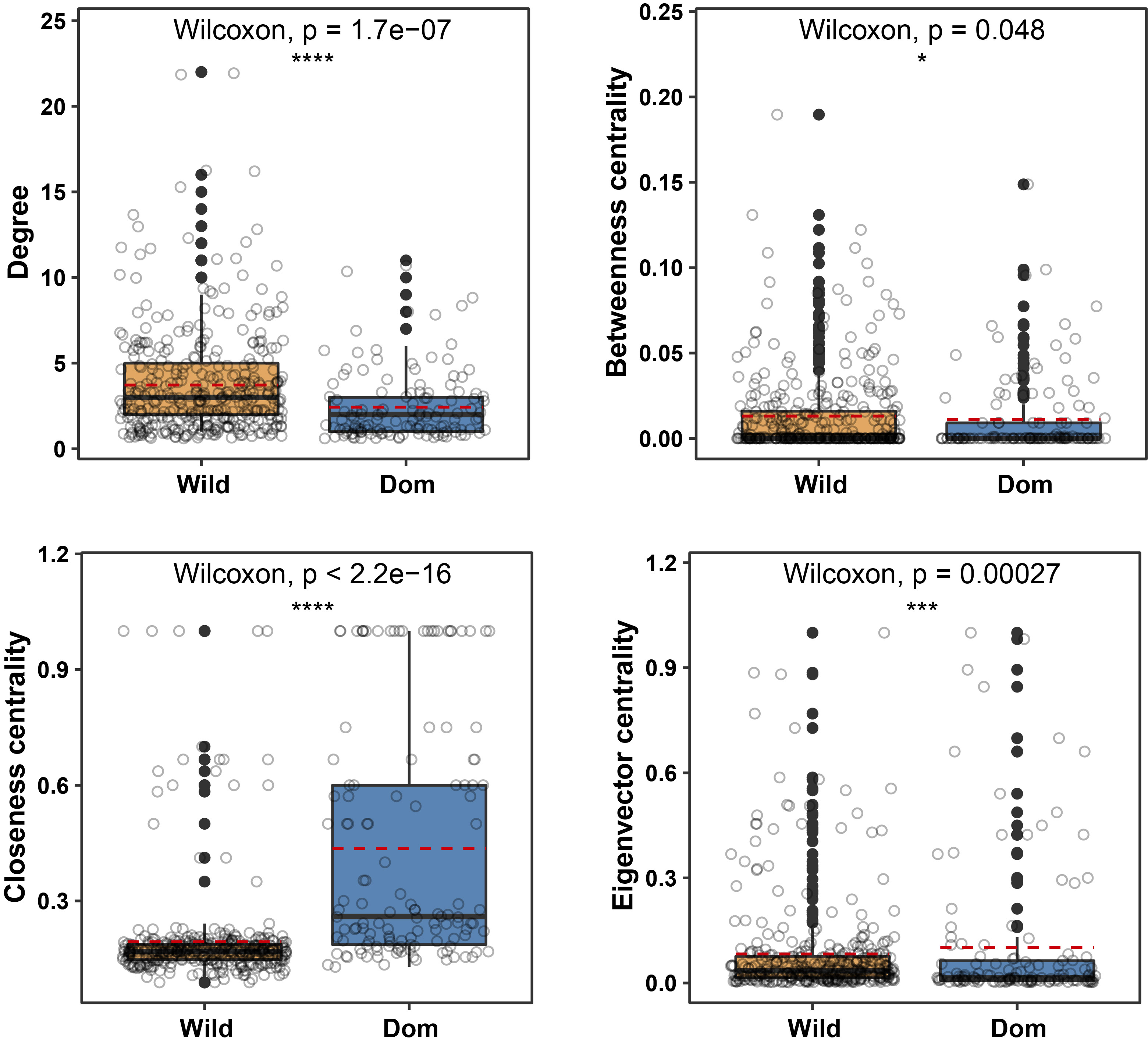


**Figure S22. Comparison on network attributes between microbial co-occurrence networks of wild and domesticated rice.** Statistically significant differences between dissimilarity distances were determined by Wilcoxon rank-sum test (****, P < 0.0001; ***, P < 0.001; **, P < 0.01; *, P < 0.05; ns, P > 0.05). The red dashed lines indicate the mean values of each centrality indices.


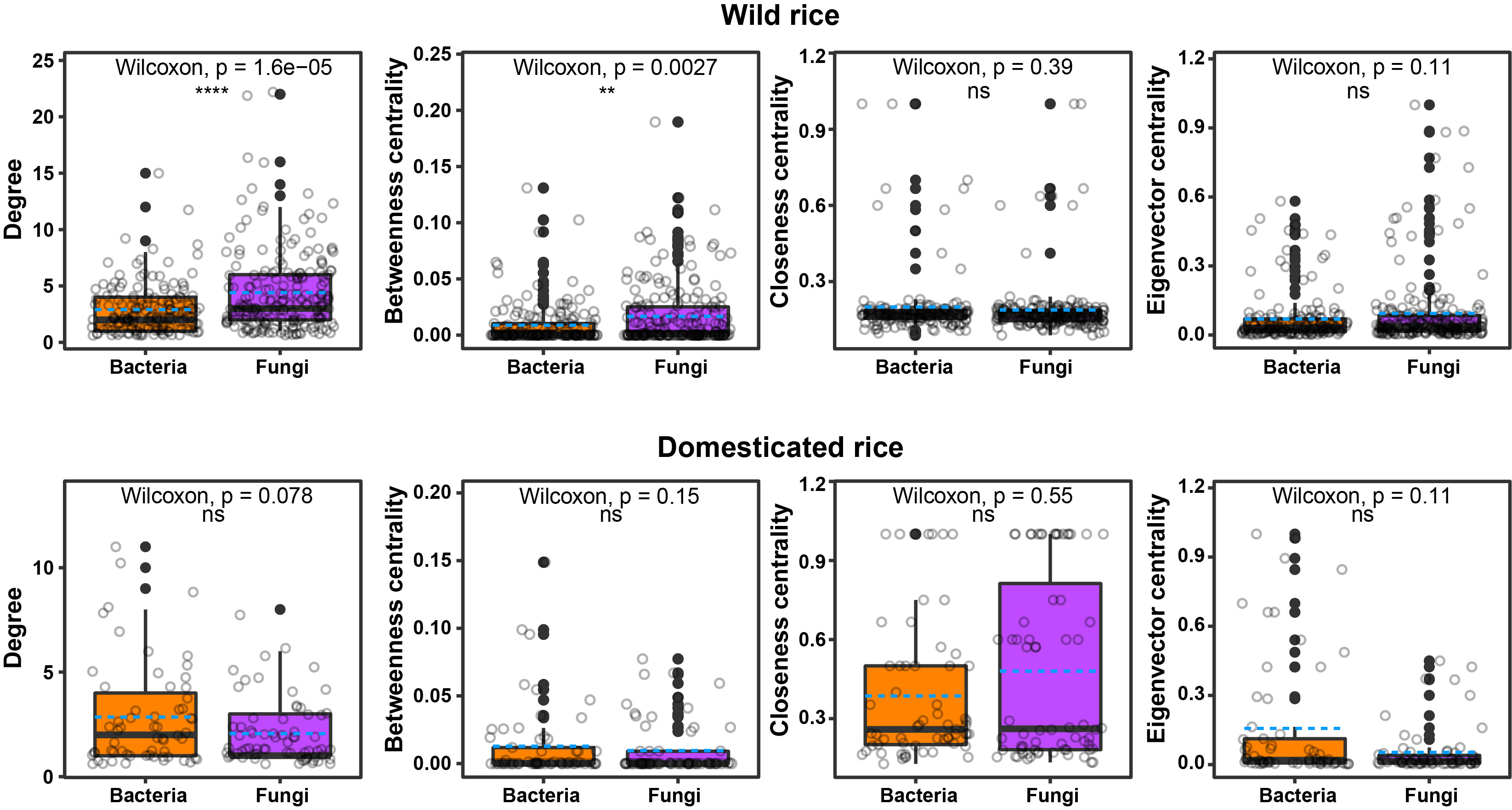


**Figure S23. Comparison on network attributes between bacterial and fungal nodes in microbial co-occurrence networks of wild and domesticated rice.** Upper and lower panels indicate co-occurrence networks of wild and domesticated rice, respectively. The blue dashed lines indicate the mean values of each centrality. Statistically significant differences between dissimilarity distances were determined by Wilcoxon rank-sum test (****, P < 0.0001; ***, P < 0.001; **, P < 0.01; *, P < 0.05; ns, P > 0.05).


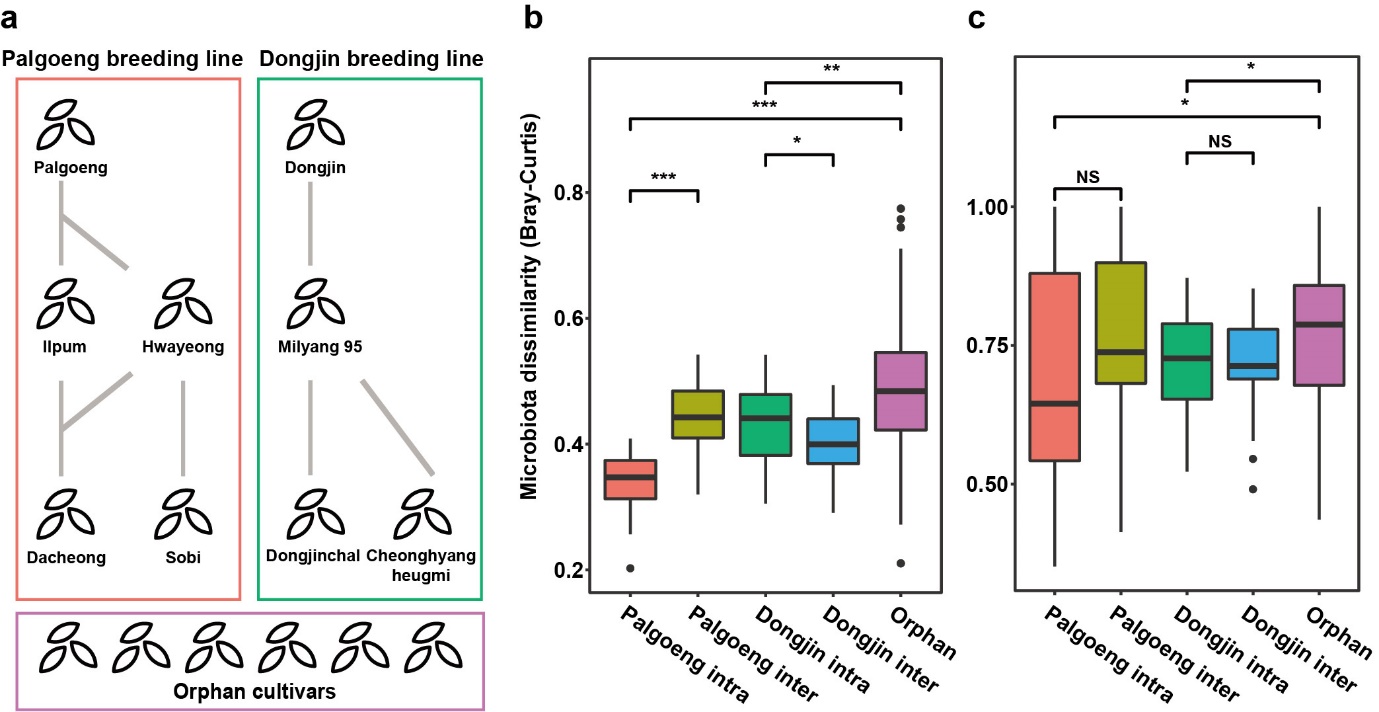


**Figure S24. Evidence of vertical transmission by comparing Bray-Curtis distances of Palgoeng and Dongjin breeding lines.** (a) Diagram showing compared groups: Palgoeng and Dongjin breeding lines, and orphan cultivars. Dissimilarity distance was calculated with Bray-Curtis distance of CSS normalized OTU table within groups and between groups (Palgoeng inter and Dongjin inter) in (b) bacterial and (c) fungal communities. Statistically significant differences between dissimilarity distances were determined by Wilcoxon rank-sum test (***, P < 0.001; **, P < 0.01; *, P < 0.05; NS, P > 0.05). Three replicates per accession were used. Microbiota dissimilarity estimated within a breeding line (dissimilarity distance between an ancestor and their descendant cultivars) was indicated as ‘intra’. Microbiota dissimilarity estimated between breeding lines (dissimilarity distance between an ancestor and descendant cultivars of other pedigree) was indicated as ‘inter’.


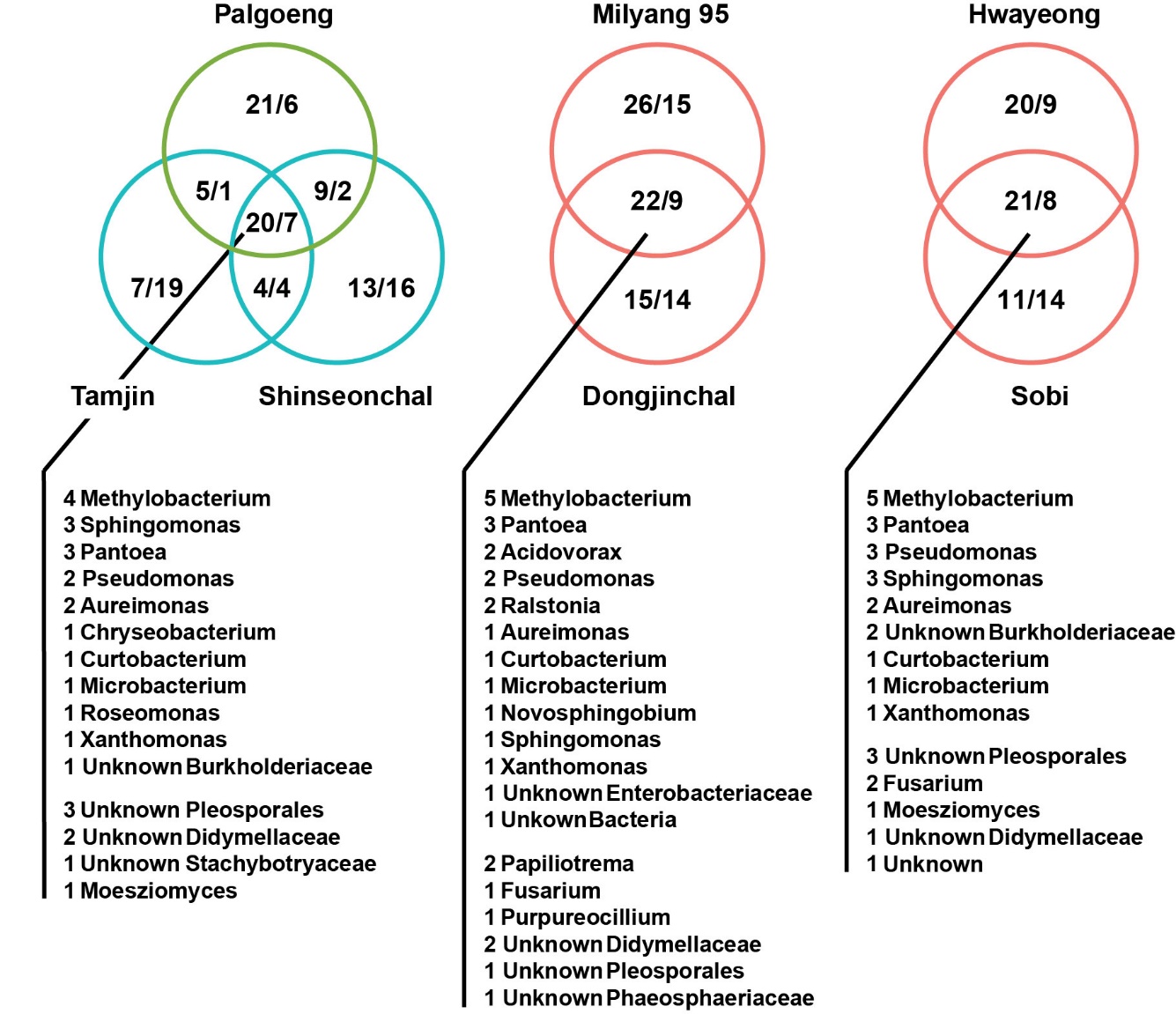


**Figure S25. Bacterial and fungal OTUs commonly detected in separate lines.** To compare taxonomical transmission tendency, we selected two first-degree lines (Milyang 95-Dongjinchal and Hwayeong-Sobi) and one common ancestor group (Palgoeng-Tamjin and Palgoeng-Shinseonchal). We showed shared OTUs in each component by presence/absence concept. The numbers in each circle mean the number of bacterial and fungal OTUs (Bacteria/Fungi).

**Figure S26**


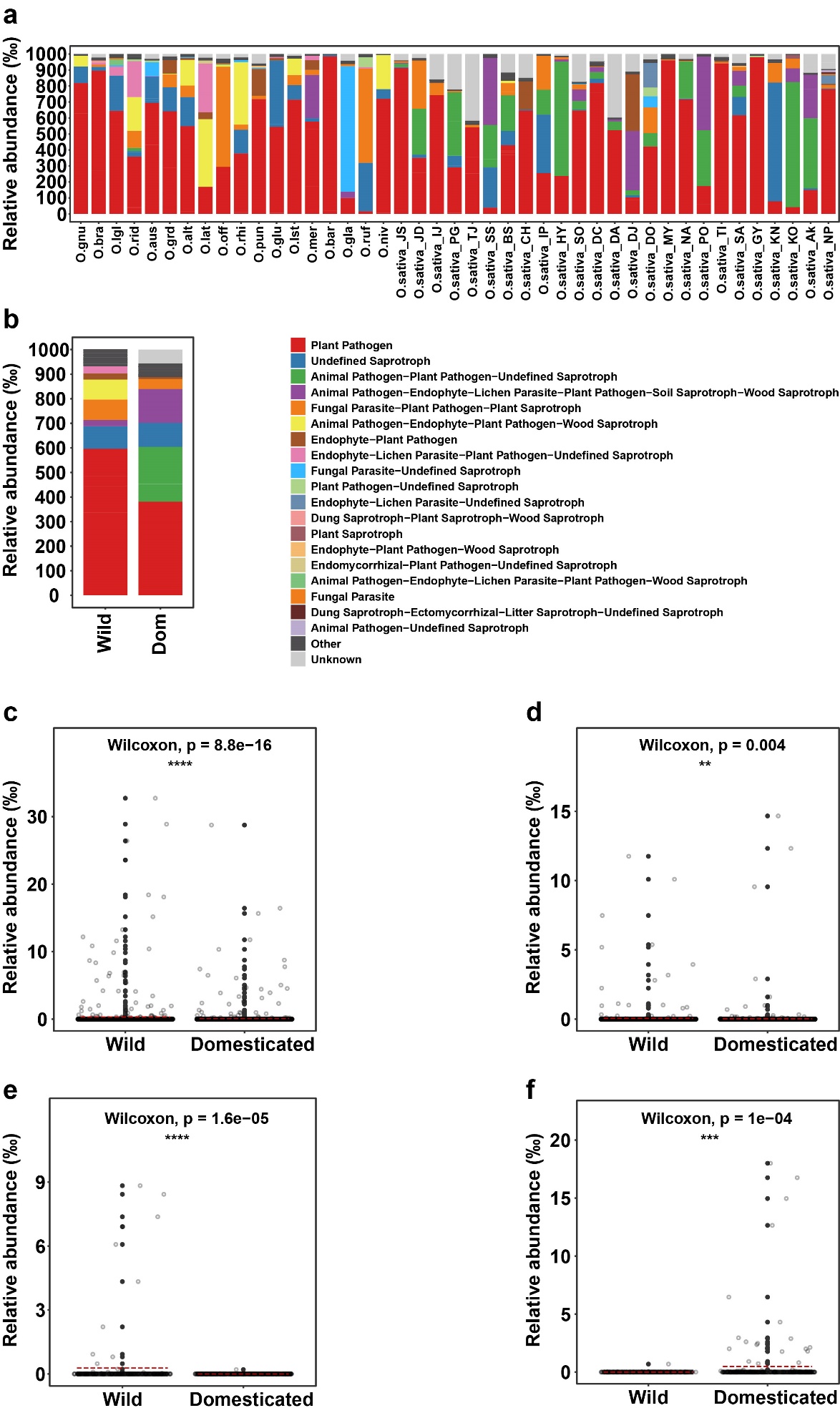


**Figure S26. Distribution of ecological guilds of fungal communities associated with wild and domesticated rice seeds.** (a) Distribution of fungal guilds in each rice accession. (b) Relative abundance of fungal guilds in the wild and domesticated rice. (c-f) Statistical analyses on the relative abundance of fungal guilds. (c) Putative plant pathogens; (d) Undefined saprotrophs; (e) Animal pathogen-Endophyte-Plant pathogen-Wood saprotroph; (f) Animal pathogen-Plant pathogen-Undefined saprotroph. The red dashed lines indicate the mean abundance of each guild. Asterisk indicates the significance based on unpaired Wilcoxon rank sum test. ****, p < 0.0001; ***, p < 0.001; **, p < 0.01; *, p < 0.05, ns (non-significant), p >0.05.

**Table S1. Abbreviation of rice accessions used for further analyses**

| **Wild rice** | | **Domesticated rice** | |
| --- | --- | --- | --- |
| **Rice accession** | **Abbreviation** | **Rice accession** | **Abbreviation** |
| *O*. *granulata* | O.gnu | *O*. *glaberrima* | O.gla |
| *O*. *brachyantha* | O.bra | Joshinryeok | O.sativa_JS |
| *O*. *longiglumis* | O.lgl | Jodongji | O.sativa_JD |
| *O*. *ridleyi* | O.rid | Iljin | O.sativa_IJ |
| *O*. *australiensis* | O.aus | Palgoeng | O.sativa_PG |
| *O*. *grandiglumis* | O.grd | Tamjin | O.sativa_TJ |
| *O*. *alta* | O.alt | Shinseonchal | O.sativa_SS |
| *O*. *latifolia* | O.lat | Boseokheugchal | O.sativa_BS |
| *O*. *officinalis* | O.off | Cheonghyangheugmi | O.sativa_CH |
| *O*. *rhizomatis* | O.rhi | Ilpum | O.sativa_IP |
| *O*. *punctata* | O.pun | Hwayeong | O.sativa_HY |
| *O*. *glumipatula* | O.glu | Sobi | O.sativa_SO |
| *O*. *longistaminata* | O.lst | Dacheong | O.sativa_DC |
| *O*. *meridionalis* | O.mer | Daean | O.sativa_DA |
| *O*. *barthii* | O.bar | Dongjin | O.sativa_DJ |
| *O*. *rufipogon* | O.ruf | Dongjinchal | O.sativa_DO |
| *O*. *nivara* | O.niv | Milyang 95 | O.sativa_MY |
|  |  | Nakdong | O.sativa_NA |
|  |  | Pungok | O.sativa_PO |
|  |  | Tongil | O.sativa_TI |
|  |  | Satbyeol | O.sativa_SA |
|  |  | Gaya | O.sativa_GY |
|  |  | Kinuhikari | O.sativa_KN |
|  |  | Koshihikari | O.sativa_KO |
|  |  | Akibare | O.sativa_AK |
|  |  | Nipponbare | O.sativa_NP |

**Table S2. Primers used in this study**

|  | **Primer** | **Sequence** |
| --- | --- | --- |
| **Bacteria** | 515F | 5’-TCGTCGGCAGCGTCAGATGTGTATAAGAGACAGGTGCCAGCMGCCGCGGTAA-3’ |
|  | 806R | 5’-GTCTCGTGGGCTCGGAGATGTGTATAAGAGACAGGGACTACHVGGGTWTCTAAT-3’ |
| **Fungi** | ITS3 | 5’-TCGTCGGCAGCGTCAGATGTGTATAAGAGACAGGCATCGATGAAGAACGCAGC-3’ |
|  | ITS4 | 5’-GTCTCGTGGGCTCGGAGATGTGTATAAGAGACAGTCCTCCGCTTATTGATATGC-3’ |

**Table S3. PCR blockers used in this study**

| **Name** | **Sequence** |
| --- | --- |
| **"mPNA" Mitochondrial Blocker** | 5'-GGCAAGTGTTCTTCGGA-3' |
| **"pPNA" Plastid Blocker** | 5'-GGCTCAACCCTGGACAG-3' |

**Table S4. Evaluation on accuracy of 5 classifier models**

| **Kingdom** | **Classifier** | **Area under ROC curve  (AUC)** | **Cross-validation  accuracy** | **Standard deviation of accuracy** |
| --- | --- | --- | --- | --- |
| ***Bacteria*** | Random forest | 0.924 | 0.8944444 | 0.1207615 |
|  | SVM | 0.897 | 0.8972222 | 0.1109567 |
|  | K-nearest neighbors | 0.786 | 0.8083333 | 0.1471028 |
|  | logistic regression | 0.669 | 0.5208333 | 0.1978591 |
|  | Naïve bayes | 0.5 | 0.5833333 | 0.06897993 |
| ***Fungi*** | Random forest | 0.889 | 0.9638889 | 0.05826716 |
|  | SVM | 0.864 | 0.9180556 | 0.056871 |
|  | logistic regression | 0.706 | 0.7805556 | 0.09550961 |
|  | K-nearest neighbors | 0.611 | 0.7666667 | 0.05430696 |
|  | Naïve bayes | 0.5 | 0.5833333 | 0.03586096 |

**References**

1. Cheng ZQ, Huang XQ, Zhang YZ, Qian J, Yang MZ, Wu CJ, Liu JF. Diversity in the content of some nutritional components in husked seeds of three wild rice species and rice varieties in Yunnan Province of China. J Integr Plant Biol. 2005;47:1260-1270.
