# Supplementary material for: Domestication of Oryza species eco-evolutionarily shapes bacterial and fungal communities in rice seed
Source: Microbiome. 2020 Feb 14;8:20. doi: 10.1186/s40168-020-00805-0 (PMC7023700; doi:10.1186/s40168-020-00805-0)
Supplement: Supplementary file 14 — Additional file 13. Pedigree of rice cultivars developed in South Korea. Information on each cultivar is gathered from Nongsaro (http://www.nongsaro.go.kr) managed by the Rural Development Administration, Korea. Based on parental relationships, the pedigree was constructed using Helium (The James Hutton Institute, version 1.17.08.14). Cultivars used in this study were indicated as red circles for Japonica and orange circles for Tongil types. Each color of the lines indicates each breeding line (green, Dongjin breeding line; brown, IR 24 breeding line; purple, Koshihikari breeding line; orange, Nipponbare breeding line; blue, Nongrim 6 breeding line; dark red, Palgoeng breeding line; black, TN1 breeding line). [file 40168_2020_805_MOESM13_ESM.pdf]

Additional file 13

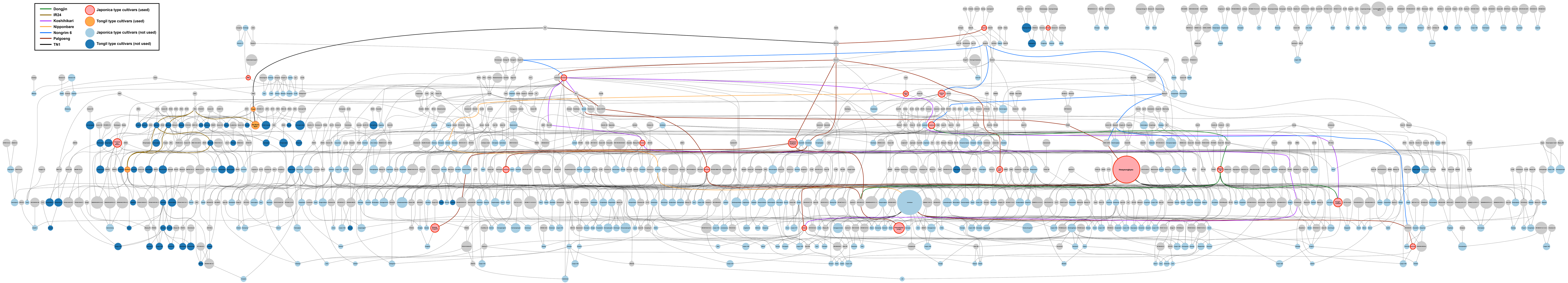

Additional file 13. Pedigree of rice cultivars developed in South Korea. Information on each cultivar is gathered from Nongsaro (http://www.nongsaro.go.kr) managed by the Rural Development Administration, Korea.

Based on parental relationships, the pedigree was constructed using Helium (The James Hutton Institute, version 1.17.08.14). Cultivars used in this study were indicated as red circles for Japonica and orange circles for Tongil types.

Each color of the lines indicates each breeding line (green, Dongjin breeding line; brown, IR 24 breeding line; purple, Koshihikari breeding line; orange, Nipponbare breeding line; blue, Nongrim 6 breeding line; dark red, Palgoeng breeding line; black, TN1 breeding line).
